# Supplementary material for: Insights into receptor structure and dynamics at the surface of living cells
Source: Nat Commun. 2023 Mar 22;14:1596. doi: 10.1038/s41467-023-37284-4 (PMC10033668; doi:10.1038/s41467-023-37284-4)
Supplement: Supplementary file 1 — Supplementary Information [file 41467_2023_37284_MOESM1_ESM.pdf]

Supplementary Information

Title

Insights into Receptor Structure and Dynamics at the Surface of Living Cells

Author list

Frederik Steiert[1,2,#], Peter Schultz[1,#], Siegfried Höfinger[3,4], Thomas D. Müller[5], Petra Schwille[1], Thomas Weidemann[1,*]

Affiliations

[1]Department of Cellular and Molecular Biophysics, Max Planck Institute of Biochemistry, Am Klopferspitz 18, 82152 Martinsried, Germany

[2]Department of Physics, Technical University Munich, 85748 Garching, Germany

[3]VSC Research Center, TU Wien, Operngasse 11 / E057-09, 1040 Wien, Austria

[4]Department of Physics, Michigan Technological University, 1400 Townsend Drive, 49931 Houghton, MI, USA

[5]Biozentrum, Julius-von-Sachs-Institut für Biowissenschaften, Lehrstuhl für Molekulare Pflanzenphysiologie und Biophysik - Botanik I, Julius-von-Sachs-Platz 2, 97082 Würzburg, Germany

[#]These authors contributed equally to this work

[*]Corresponding author. Email: weidemann@biochem.mpg.de

This Supplementary Information includes:

Supplementary Figures S1 to S29

Supplementary Tables S1 to S8

Other Supplementary Materials for this manuscript include the following:

Supplementary Movies S1 to S9

Computational Biology Toolkit (SI_comp_bio.zip)

Source Data file

Supplementary Figures

Supplementary Figure 1 Steiert, Schultz et al.

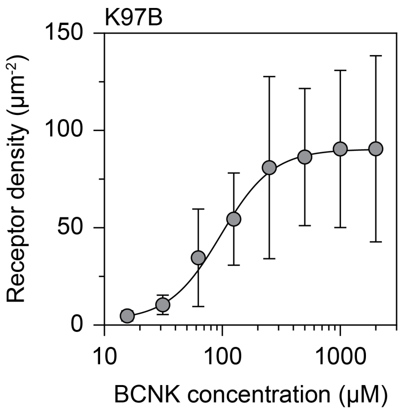

Supplementary Fig. 1. Effect of BCNK concentration on GCE-mediated receptor expression. HEK293T cells transfected with the receptor mutant K97B were incubated with varying concentrations of BCNK in the growth medium. The surface plasma membrane was stained with Cy5-NHS and the cells were imaged by confocal laser scanning microscopy. Note that membranes were detected in the Cy5-NHS channel. The receptor density is based on eGFP fluorescence at the plasma membrane after image segmentation and intensity calibration using the scripts of the BCRI approach neglecting a second color channel. Particle numbers were transformed into receptor density using the geometry of the observation volume as determined by FCS calibration. Receptor densities were fitted to the Hill equation (black line, concentration at half response: 101 µM, hill coefficient: 1.9). For each concentration, seven large-scale images (213 µm x 213 µm) were recorded containing in total between 400 and 500 randomly sampled cells. Markers and error bars represent mean ± SD of the average receptor density in the seven images. Descriptive statistics and details about number of measurements in Supplementary Table 1. Experiment was performed once. Source data are provided as a Source Data file.

Supplementary Figure 2 Steiert, Schultz et al.

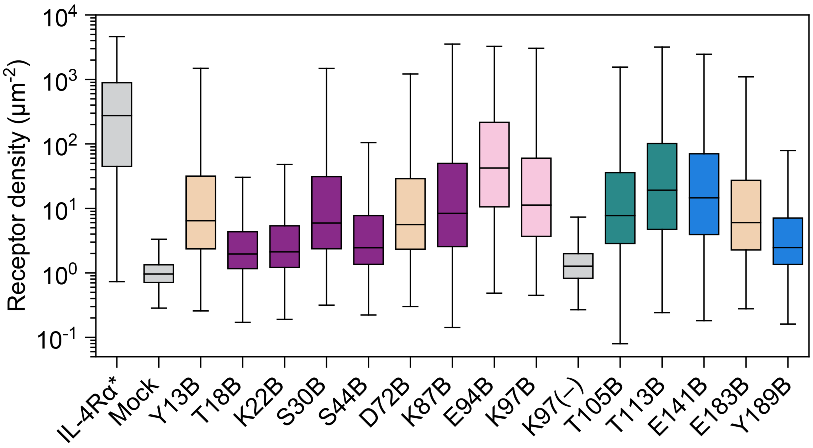

Supplementary Fig. 2. GCE expression quantified by brightness-calibrated imaging (BCRI). Comparison of GCE-mediated expression of the receptor mutants (color code according to Fig. 1a; mature numbering; Uniprot P24394-1) relative to IL-4Rα* under control of the same CMV promoter. Receptor mutants were expressed in the presence of 0.5 mM BCNK. As negative controls, transfection was either performed without plasmid DNA (Mock) or K97B was expressed without BCNK in the growth medium (K97(–)). Imaging was performed 18 h post-transfection. Note that membranes were detected in the Cy5-NHS channel. The receptor density is based on eGFP fluorescence at the plasma membrane after image segmentation and intensity calibration using the scripts of the BCRI approach neglecting a second color channel. Particle numbers were transformed into receptor density using the geometry of the observation volume as determined by FCS calibration. Box-and-whisker plots indicate first and third quartile (box), median (horizontal line), and 1.5 times the interquartile range (whiskers). Descriptive statistics and details about number of measurements in Supplementary Table 2. Experiments were performed three (IL-4Ra*, K87B) or two (else) times. Source data are provided as a Source Data file.

Supplementary Figure 3 Steiert, Schultz et al.

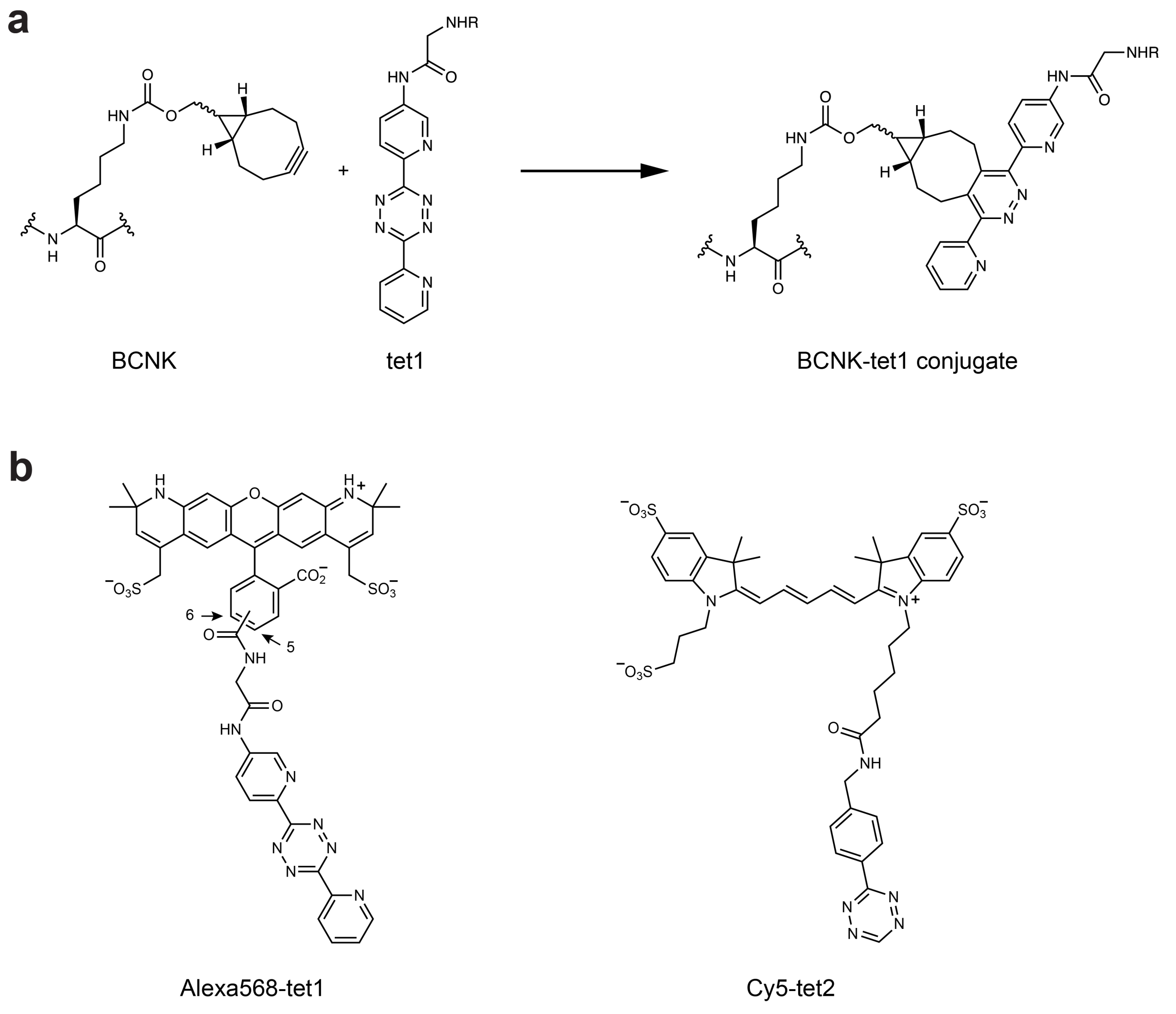

Supplementary Fig. 3. Inverse electron-demand Diels-Alder cycloaddition (iEDDAC) of tetrazine derivatives to the non-canonical amino acid (ncAA) side chain. (**a**) Chemical structure of the ncAA bicyclo[6.1.0]nonyne-lysine (BCNK) as used for GCE expression of receptor mutants containing a stop-codon in the coding sequence. The alkyne bond of the terminal ring served as a dienophile for covalent coupling of tetrazine derivatives. (**b**) Chemical structure of fluorescent tetrazines used in this study [1,2].

Supplementary Figure 4 Steiert, Schultz et al.

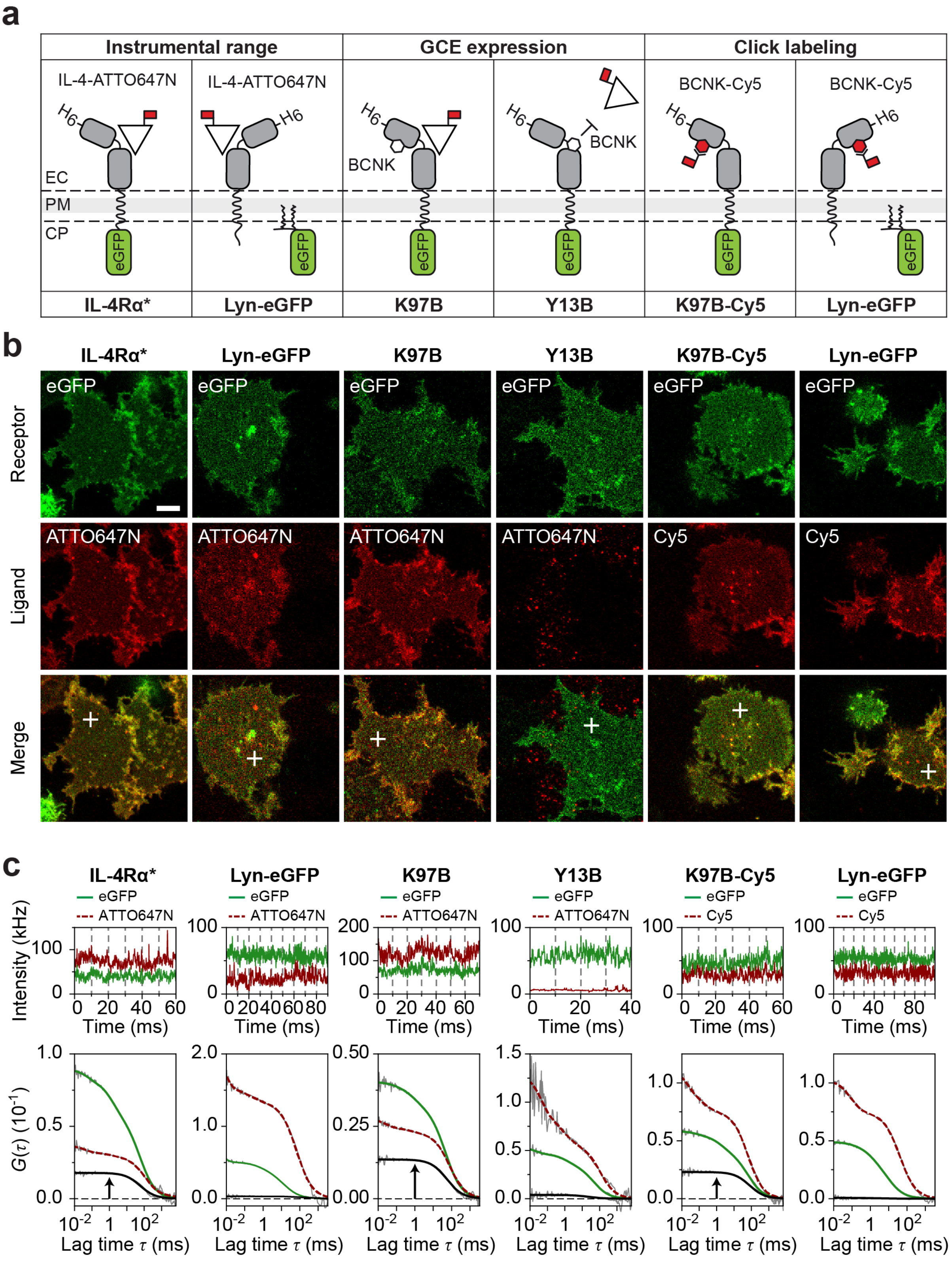

Supplementary Fig. 4. Legend continued on next page.

Supplementary Fig. 4. FCCS in living cells to characterize click labeling. (**a**) Cartoon illustrating the concept of FCCS pairwise experiments: (1.) Instrumental range for the 488 nm/633 nm setting was assessed with specific, saturated ligand binding (IL-4-ATTO647N) to IL-4Rα* *vs.* Lyn-eGFP as a non-accessible membrane marker at the inner leaflet of the plasma membrane. (2.) GCE expression addressed the structure-function-relationship of IL-4Rα* as demonstrated by the ligand binding receptor mutant K97B vs. the neutralized receptor mutant Y13B, where the BCNK blocks the binding epitope, and (3.) Click labeling demonstrates co-diffusion of the click label with intracellular eGFP that is fused to IL-4Rα* *vs.* eGFP attached to the independently diffusing Lyn-eGFP. (**b**) Representative examples for confocal images of the bottom membrane of the measured cells. Receptors, ligands and Cy5-tetrazine conjugates are homogenously distributed. Crosses indicate typical positions for single-point FCCS measurements. Scale bar represents 5 µm. (**c**) Intensity traces of both color channels (receptors green, ligand red) and the corresponding correlation functions. Finite cross-correlation between the color channels (black) indicates co-diffusion of both labels (arrows). Experiments were performed once (Lyn-eGFP, Y13B) or twice (else). Source data are provided as a Source Data file.

Supplementary Figure 5 Steiert, Schultz et al.

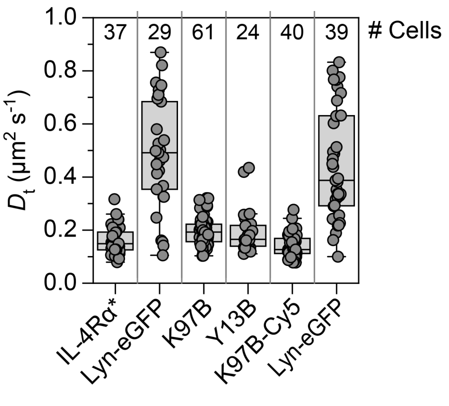

Supplementary Fig. 5. Lateral diffusion in the plasma membrane. Translational diffusion coefficients D_t based on autocorrelation functions of the eGFP channel in FCCS measurements. Since lateral diffusion is dominated by membrane-spanning domains, the measured diffusion coefficients were independent of different labels or the position of BCNK in the extracellular domain. Lyn-eGFP attached to the inner leaflet shows faster and more heterogeneous diffusion. Box-and-whisker plots indicate first and third quartile (box), median (horizontal line), and 1.5 times the interquartile range (whiskers). Experiments were performed once (Lyn-eGFP, Y13B) or twice (else). Source data are provided as a Source Data file.

Supplementary Figure 6 Steiert, Schultz et al.

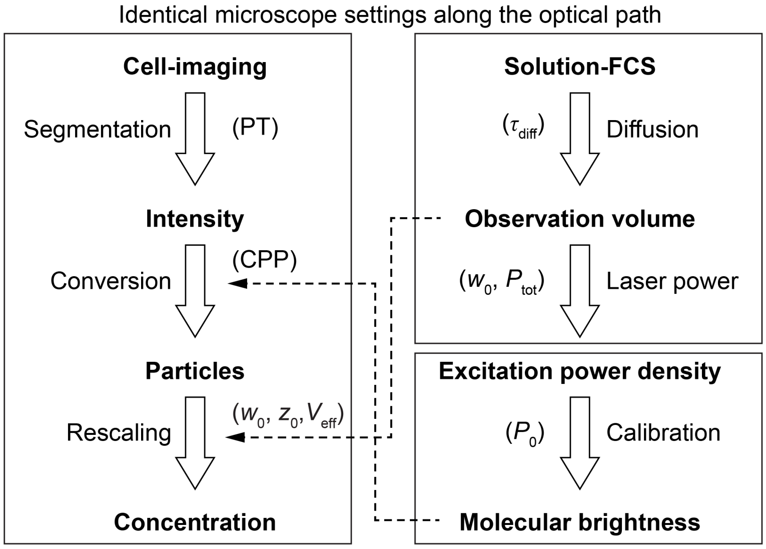

Supplementary Fig. 6. Workflow of brightness-calibrated ratiometric imaging (BCRI). The experimental procedure combines three lines of data input that were performed for each experimental session: Confocal live-cell imaging of fluorescently-labeled cells (left panel), FCS measurements to determine the molecular brightness of the imaged labels (top right panel), and determination of the total laser power in each color channel (lower right panel). For imaging and FCS calibration, identical microscope settings are crucial to combine the results. The molecular brightness of the labels can be used to convert pixel counts in confocal images to particle numbers and concentration. Abbreviations: pixel dwell time (PT), counts per particle (CPP), waist of the observation volume in y,x (w_0), dimension of the observation volume along the optical axis (z_0), effective three-dimensional volume (V_{eff}), diffusion time (τ_{diff}), total laser power (P_{tot}), excitation power density (P_0).

Supplementary Figure 7 Steiert, Schultz et al.

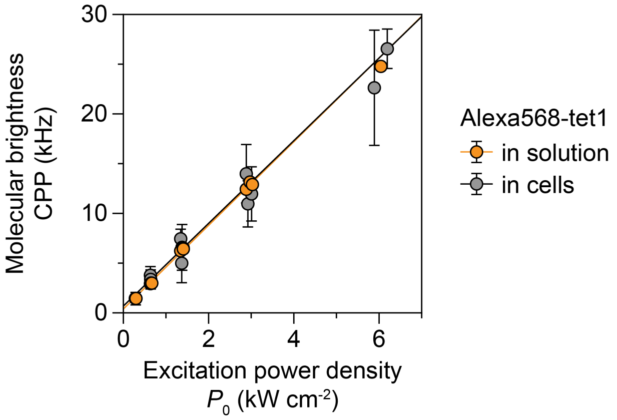

Supplementary Fig. 7. Molecular brightness measured by single-color fluorescence correlation spectroscopy (FCS). The molecular brightness of Alexa568-tet1 reflected by the average counts per particle (CPP) in the confocal observation volume was measured by single-color FCS either in free solution (orange circles) or at the bottom membrane of HEK293T cells after click labeling of the receptor mutant K97B (gray circles). To match conditions at the plasma membrane, Alexa568-tet1 in solution was pre-incubated with excess amounts of reactive BCNK for conjugation. Linear regression indicates that the molecular brightness of the fluorophore Alexa568 is the same under both conditions. Laser irradiance P_0 was determined from the total laser power P_{tot} and the beam waist radius w_0 of the 561 nm laser line. Markers and error bars represent the mean ± SD of three positions in the measurement chamber (in solution) or of 5 to 24 cells (precise n numbers provided in the source data). Experiments were performed three (in solution) or four (in cells) times. Source data are provided as a Source Data file.

Supplementary Figure 8 Steiert, Schultz et al.

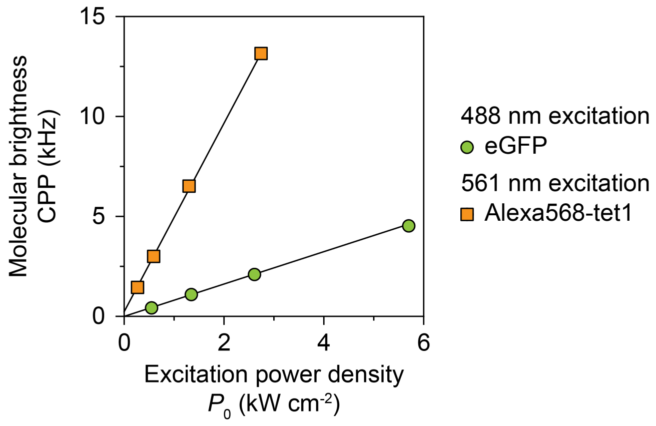

Supplementary Fig. 8. Molecular brightness reference of the labels eGFP and Alexa488-tet1. Average counts per particle (CPP) in the confocal observation volume was measured by single-color FCS in dilute solutions of recombinant eGFP and Alexa568-tet1 coupled to BCNK for a range of excitation power densities. For both fluorophores, the CPP scales linearly with the laser irradiance (linear fit, black). The increased slope of Alexa568-tet1 is related to superior performance of the Alexa568 fluorophore in terms of photon yield. Laser irradiance P_0 was determined from the total laser power P_{tot} and the beam waist radius w_0 of the respective laser line. Source data are provided as a Source Data file.

Supplementary Figure 9 Steiert, Schultz et al.

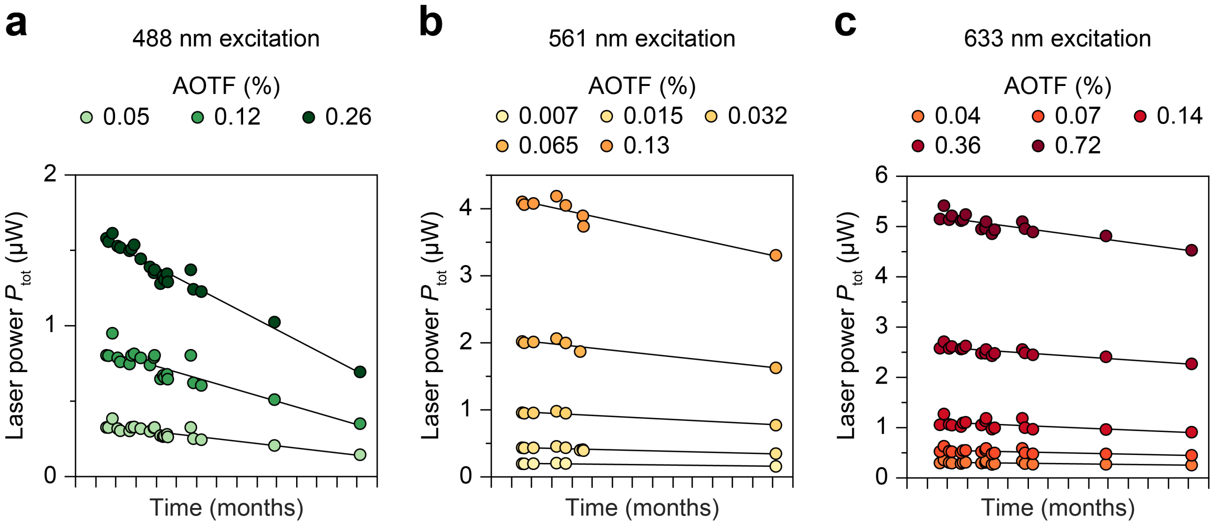

Supplementary Fig. 9. Monitoring laser power during the project. The total laser power P_{tot} of (**a**) 488 nm, (**b**) 561 nm, and (**c**) 633 nm laser lines were measured with a power meter behind the objective lens for a range of transmission percentages of the acousto-optic tunable filter (AOTF, LSM780, Carl Zeiss Microscopy) used in live-cell imaging. For all three channels, a decreasing power output over time can be observed due to aging (linear fitting, back). Each marker represents the average P_{tot} of power measurements taken before and after imaging sessions on different experimental days. Source data are provided as a Source Data file.

Supplementary Figure 10 Steiert, Schultz et al.

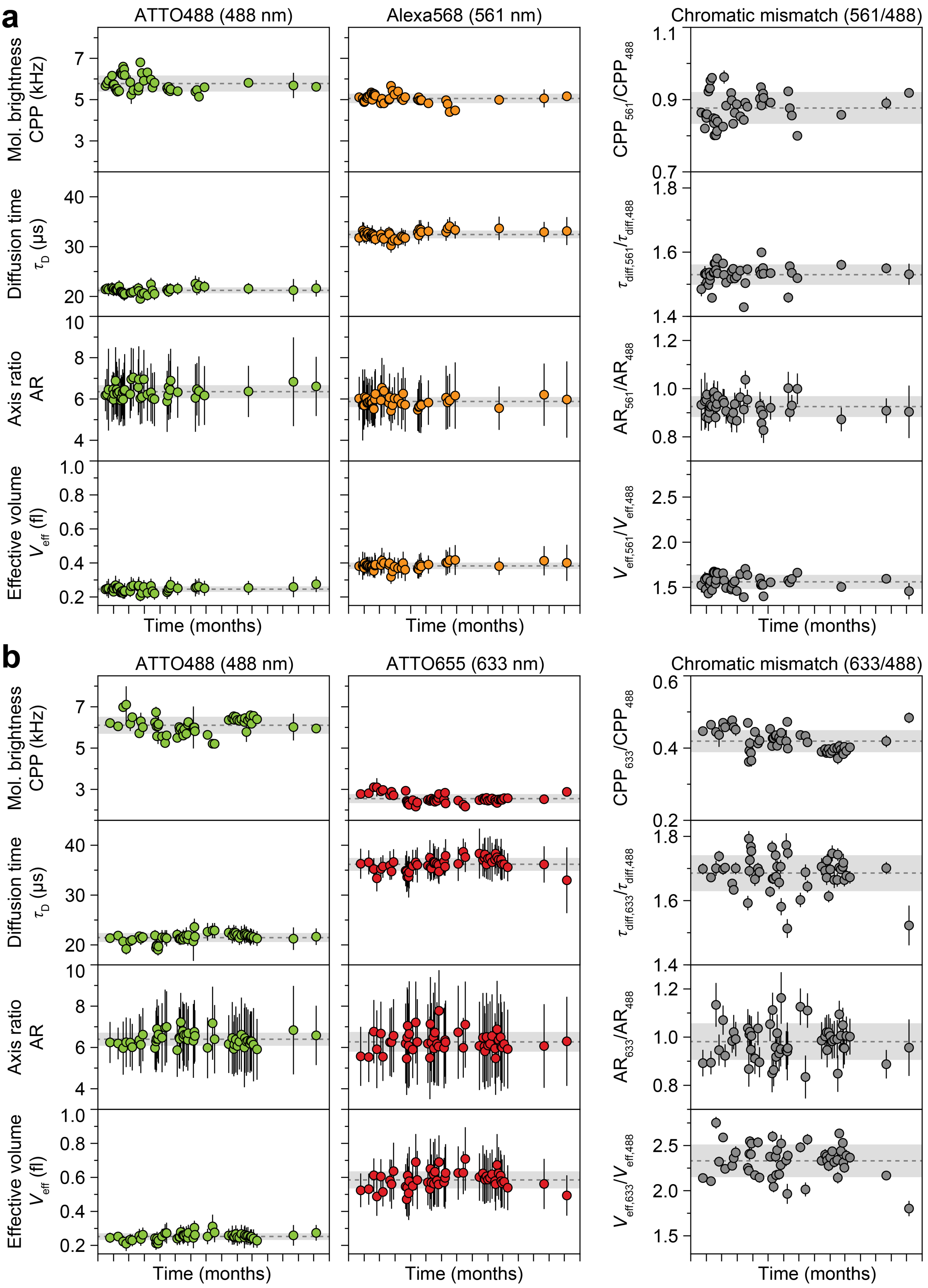

Supplementary Fig. 10. Legend continued on next page.

Supplementary Fig. 10. Monitoring optical performance for brightness-calibrated ratiometric imaging (BCRI). Results of single-color FCS calibrations in solution for (**a**) pairwise imaging of the 488 nm/561 nm channels using the calibration dyes ATTO488 (left column) and Alexa568 (middle column) and (**b**) pairwise imaging of the 488 nm/633 nm channels using the calibration dyes ATTO488 (left column) and ATTO655 (middle column). Fitting to a model function assuming unimpaired three-dimensional diffusion provides parameters that characterize the confocal observation volume (diffusion time, axis ratio, and effective volume), as well as the molecular brightness as reflected by the average counts per particle (CPP) in the observation volume. Since a ratio is considered for BCRI, the ratio of the respective parameters is also displayed (column on the right). Note that the CPPs of the calibration dyes, which were corrected for decreasing laser power output (*cf.* Supplementary Fig. 9), were not used for BCRI. Markers and error bars represent the mean ± SD of typically 18 runs with 60 s acquisition time each. Precise values for the number of runs at each date are provided in the source data. Source data are provided as a Source Data file.

Supplementary Figure 11 Steiert, Schultz et al.

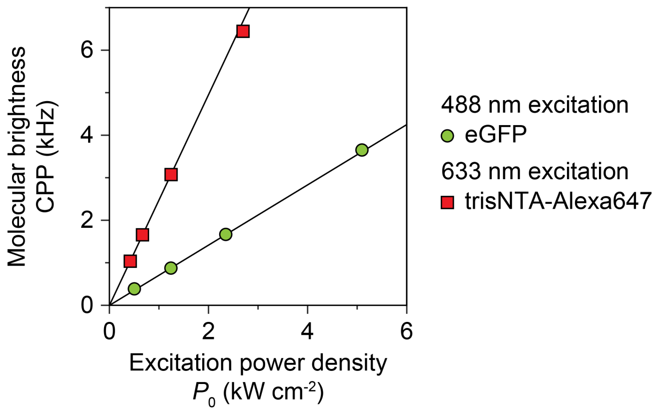

Supplementary Fig. 11. Molecular brightness reference of the labels eGFP and trisNTA-Alexa647. Average counts per particle (CPP) in the confocal observation volume was measured by single-color FCS in dilute solutions of recombinant eGFP (PBS, pH7.4) and trisNTA-Alexa647 (air buffer) for a range of excitation power densities. For both fluorophores, the CPP scales linearly with laser irradiance (linear fit, black). The increased slope of trisNTA-Alexa647 is related to superior performance of the Alexa647 fluorophore in terms of photon yield. Laser irradiance P_0 was determined from the total laser power P_{tot} and the beam waist radius w_0 of the respective laser line. Source data are provided as a Source Data file.

Supplementary Figure 12 Steiert, Schultz et al.

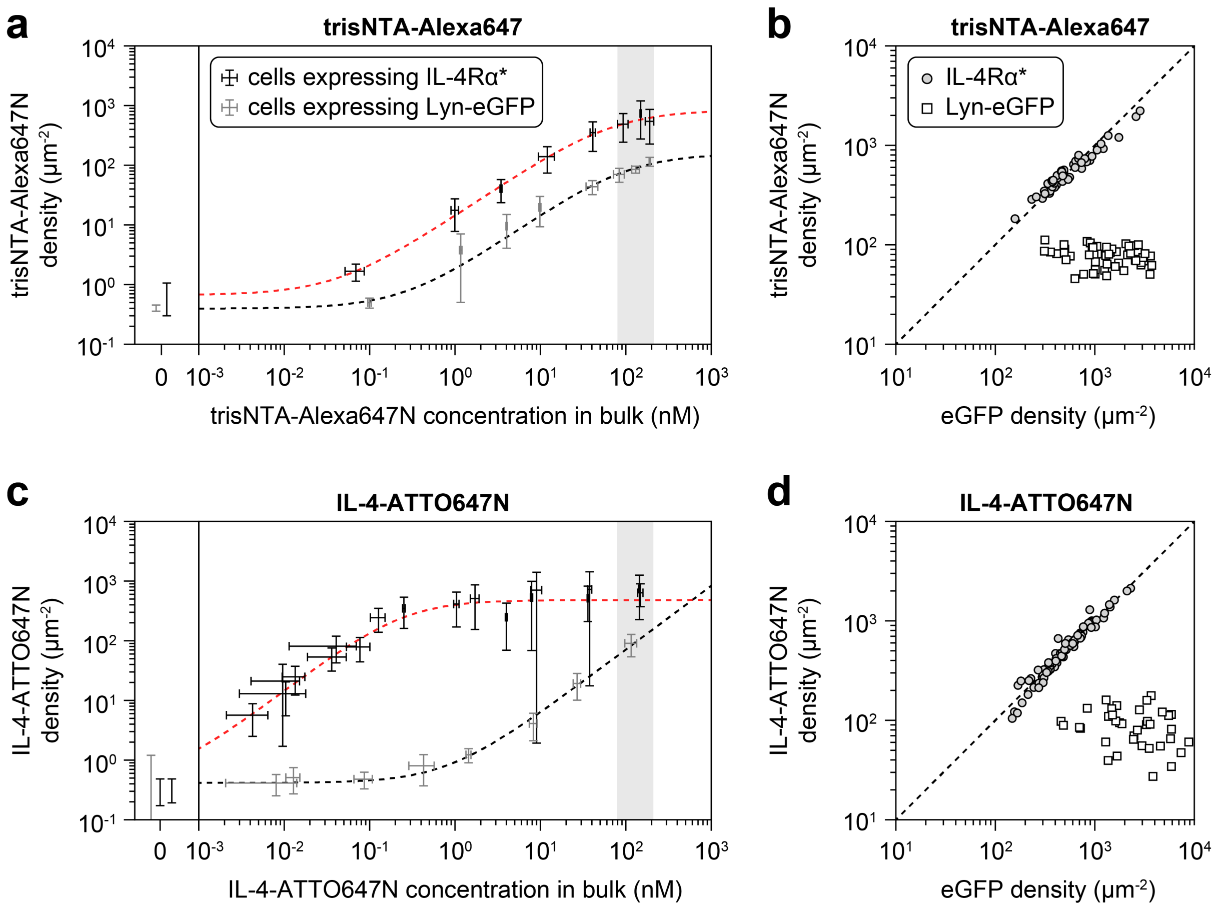

Supplementary Fig. 12. Discriminating specific and non-specific binding of the His-tag tracer trisNTA-Alexa647 and the natural ligand IL-4-ATTO647N at the cell surface. HEK293T cells were transfected with IL-4Rα*, containing a His-tag at the extracellular N-terminus and C-terminal eGFP, or control cells expressing Lyn-eGFP, a cytoplasmic plasma membrane marker inaccessible from the bulk. After incubation with decreasing amounts of trisNTA-Alexa647 (top row) or IL-4-ATTO647N (bottom row), cells were imaged and analyzed by BCRI. (**a, c**) Cell-bound ligand density plotted against the concentration of free ligand in the supernatant as determined by FCS. At higher ligand concentrations, significant ligand binding occurs even for control cells indicating hidden, probably charge-mediated, binding sites in the native plasma membrane. (**b**, **d**) Correlation analysis for high concentrations of ligand (grey shaded range in **a**, **c**). Ligand binding to the plasma membrane and receptor expression levels are highly correlated when expressing IL-4Rα* (filled circles) but uncorrelated for control cells expressing Lyn-eGFP (unfilled squares). Dashed lines represent fits to the Hill equation (**a**, **c**) or the relation expected for a 1:1 binding reaction (**b**, **d**). Markers and error bars represent mean ± SD of 13–155 cells (y-axis, precise number of cells for each concentration provided in the source data) or 18 FCS runs (x-axis). Experiments were performed once (a) or twice (c). Source data are provided as a Source Data file.

Supplementary Figure 13 Steiert, Schultz et al.

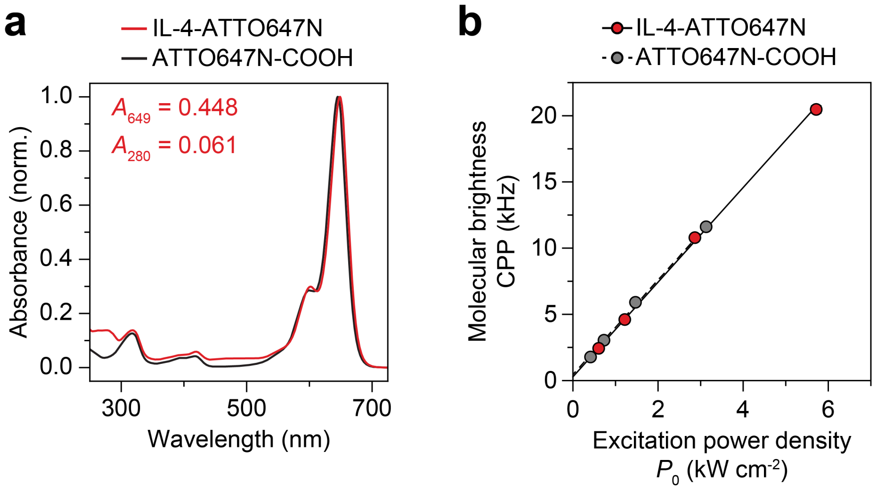

Supplementary Fig. 13. Characterizing IL-4-ATTO647N. (**a**) Peak-normalized absorption spectra of IL-4-ATTO647N (red line) and ATTO647N-COOH (black line) as used to determine the degree of labeling (DOL = 68%). (**b**) Molecular brightness reflected by the average counts per particles (CPP) of the fluorophore ATTO647N in the confocal observation volume measured by single-color FCS for the free carboxylic acid form (red circles) and coupled to IL-4 (grey circles) in comparison. The molecular brightness of ATTO647N is not affected by maleimide-coupling. The CPP scales linearly with laser irradiance (linear fit, black). The laser irradiance P_0 was determined from the total laser power P_{tot} and the beam waist radius w_0 of the respective laser line. Source data are provided as a Source Data file.

Supplementary Figure 14 Steiert, Schultz et al.

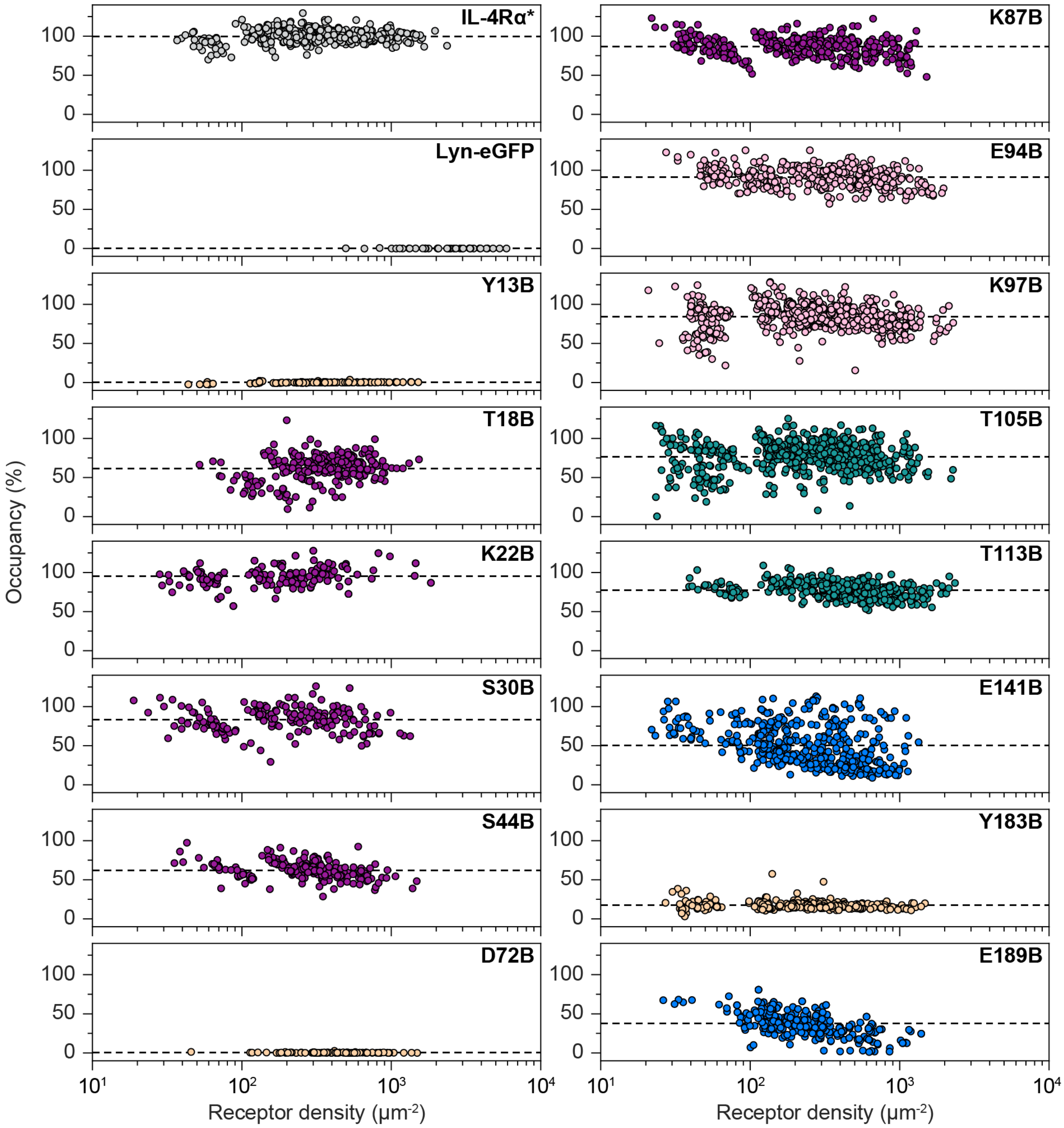

Supplementary Fig. 14. Cellular distributions of receptor occupancy resolved with brightness-calibrated ratiometric imaging (BCRI). Quantification of the ligand binding capacity of IL-4Rα*, Lyn-eGFP and BCNK-bearing receptor mutants (color code according to Fig. 1a; mature numbering; Uniprot P24394-1) in the presence of 10 nM IL-4-ATTO647N in the supernatant. Particle numbers were transformed into receptor density using the geometry of the confocal observation volume as determined by FCS calibration. Each data point represents a cell-wise average; the population average is indicated with a dashed line. Descriptive statistics and details about number of measurements in Supplementary Table 3. Source data are provided as a Source Data file.

Supplementary Figure 15 Steiert, Schultz et al.

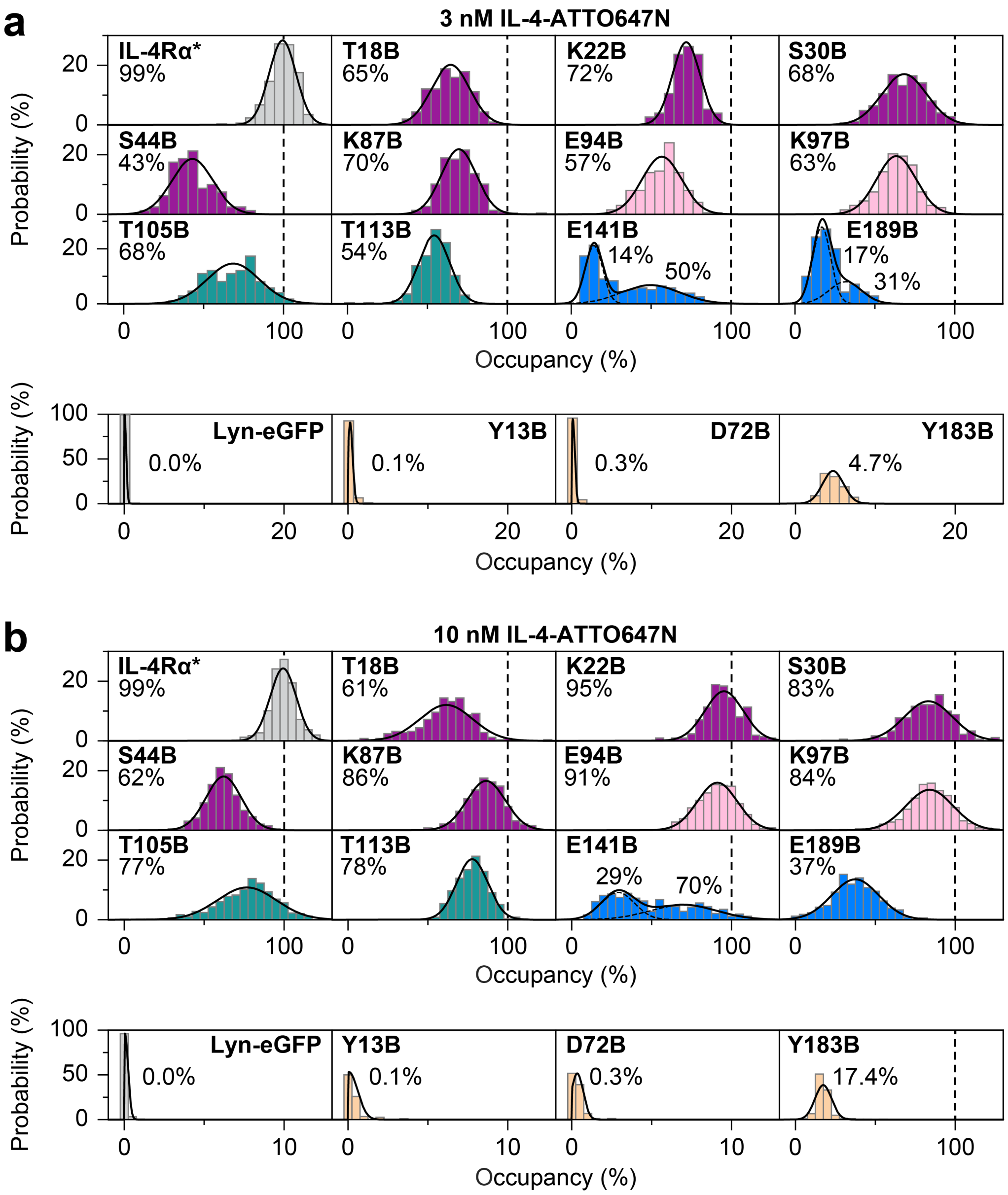

Supplementary Fig. 15. Ligand binding to BCNK-bearing receptor mutants assessed with brightness-calibrated ratiometric imaging (BCRI). The ligand binding capacity of IL-4Rα*, Lyn-eGFP and BCNK-bearing receptor mutants (color code according to Fig. 1a; mature numbering; Uniprot P24394-1) was probed with BCRI at (**a**) 3 nM and (**b**) 10 nM IL-4-ATTO647N. For each mutant, histograms built on 80–600 cell averages were plotted together with the normal distribution based on the mean and standard deviation of the data sets (black lines). In case of multimodal distributions (E141B and E189B), probability functions were estimated with a two-component Gaussian mixture model. Descriptive statistics and details about number of measurements are in Supplementary Table 3 and Supplementary Table 4. Source data are provided as a Source Data file.

Supplementary Figure 16 Steiert, Schultz et al.

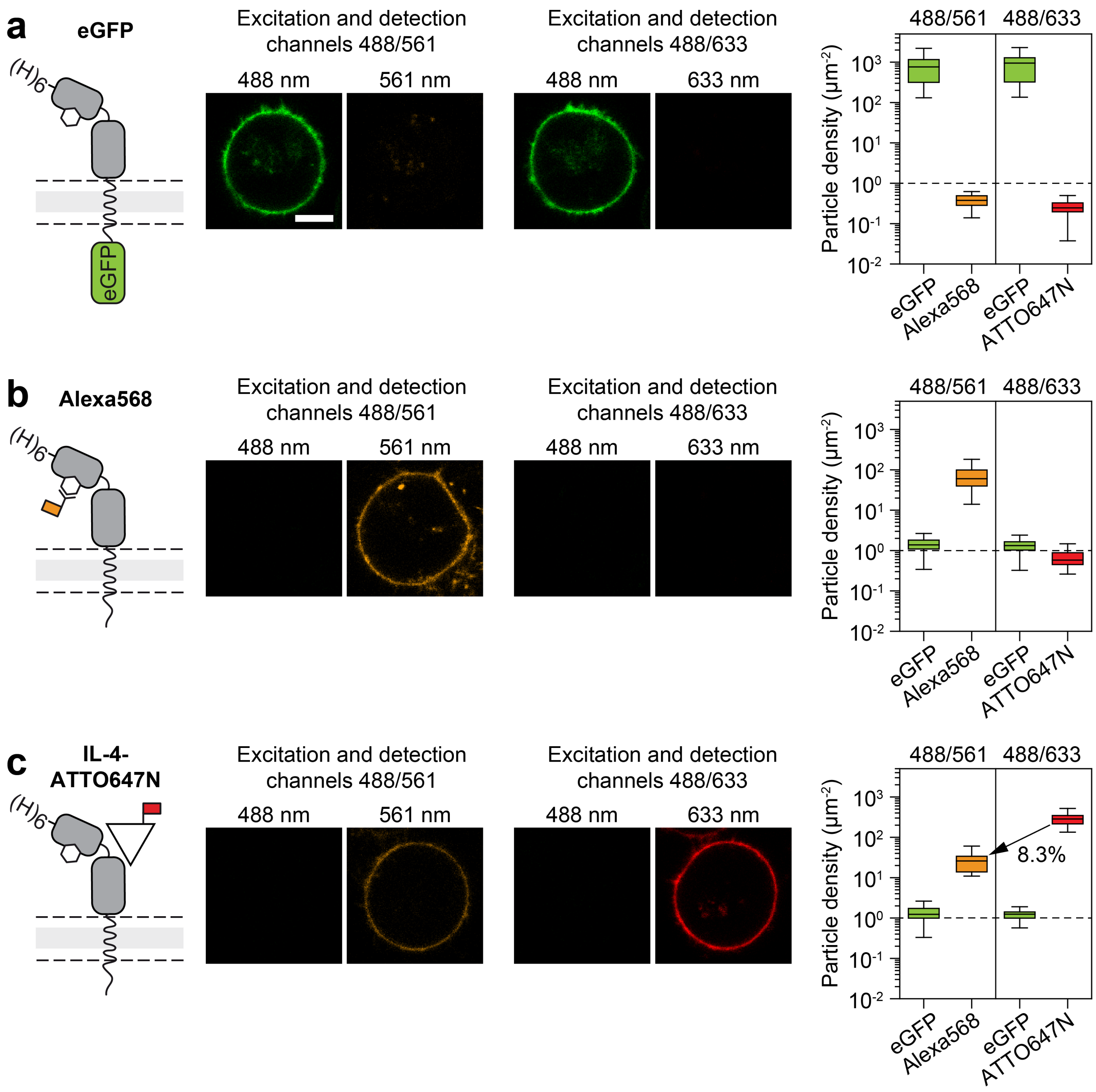

Supplementary Fig. 16. Brightness-calibrated ratiometric imaging (BCRI) combining three color channels in two pairwise configurations. Cross-excitation and spectral crosstalk was assessed by imaging single color control cells expressing (**a**) eGFP, (**b**) IL-4Rα* click-labeled by Alexa568-tet1, and (**c**) IL-4Rα* occupied by the natural ligand IL-4-ATTO647N (cartoons, left column). For each labeling condition, cells were imaged with alternating 488 nm/561 nm and 488 nm/633 nm excitation (fluorescent cross-sections, middle column), and corresponding particle densities were determined by BCRI (box plots, right column). eGFP associated signals did not spill into the red-shifted channels in either configuration. Only the far-red fluorophore ATTO647N, produces significant false signal (8.3%) in the 561 nm channel of the click-label due to cross-excitation (arrow). Box-and-whisker plots indicate first and third quartile (box), median (horizontal line), and 1.5 times the interquartile range (whiskers) of 46 (a), 83 (b) or 44 cells (c) per condition. Experiment was performed once. Scale bar represents 5 µm. Source data are provided as a Source Data file.

Supplementary Figure 17 Steiert, Schultz et al.

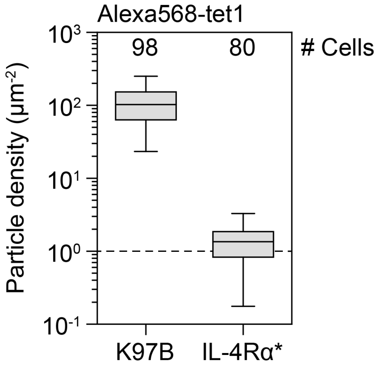

Supplementary Fig. 17. Surface density and specificity of click labeling assessed with brightness-calibrated ratiometric imaging (BCRI). Estimation of two-dimensional surface densities in the plasma membrane by considering the confocal observation volume in the 568 nm channel. Quantifying a typical set of hand-selected cells for the receptor mutant K97B ($n = 98$ cells), returned on average ~118 ± 76 Alexa568-tet1 particles per μm^2. Under the same conditions, mock-labeled control cells expressing IL-4Rα* ($n = 80$ cells) show about one particle per μm^2 (dashed line). Relative to the total pool of receptors present at the plasma membrane as determined from the eGFP signal, the Alexa568-tet1 particle densities correspond to click efficiencies of 18.0% ± 2.7% and 0.2% ± 0.1%, respectively. These numbers emphasize the high level of specificity of iEDDAC-mediated click labeling. Box-and-whisker plots indicate first and third quartile (box), median (horizontal line), and 1.5 times the interquartile range (whiskers). Experiment was performed once. Source data are provided as a Source Data file.

Supplementary Figure 18 Steiert, Schultz et al.

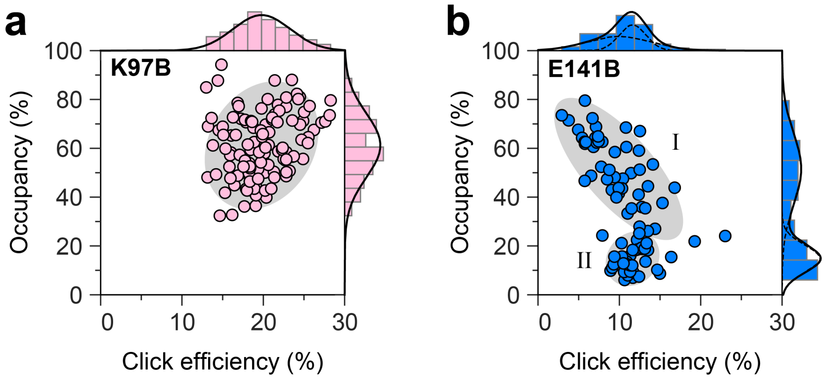

Supplementary Fig. 18. Interdependence of click labeling and ligand binding assessed by three-channel brightness-calibrated ratiometric imaging. Cells expressing (**a**) K97B or (**b**) E141B (color code according to Fig. 1a; mature numbering; Uniprot P24394-1) were click-labeled with Alexa568-tet1 and then incubated with the natural ligand IL-4-ATTO647N. Scatter plots of the average fractions of labeled receptors for each color channel show that receptor occupancy and click efficiency are mutually independent for the K97B mutant ($n = 119$ cells), whereas click labeling has negative effects on the IL-4 binding capability for the E141B mutant ($n = 85$ cells, population I). For a small population of click-labeled E141B receptor mutants, ligand binding is even destroyed (population II). The data suggests conformational rearrangements in the vicinity of the activation loop that convey allosteric effects on the ligand binding epitope [3]. Gray ellipses represent fitted probability distributions (2σ width) based on Gaussian mixture modeling with one (K97B) or two (E141B) components. Marginal probability distributions and histograms of click efficiency and receptor occupancy are displayed on top and on the right, respectively. Experiment was performed once. Source data are provided as a Source Data file.

Supplementary Figure 19 Steiert, Schultz et al.

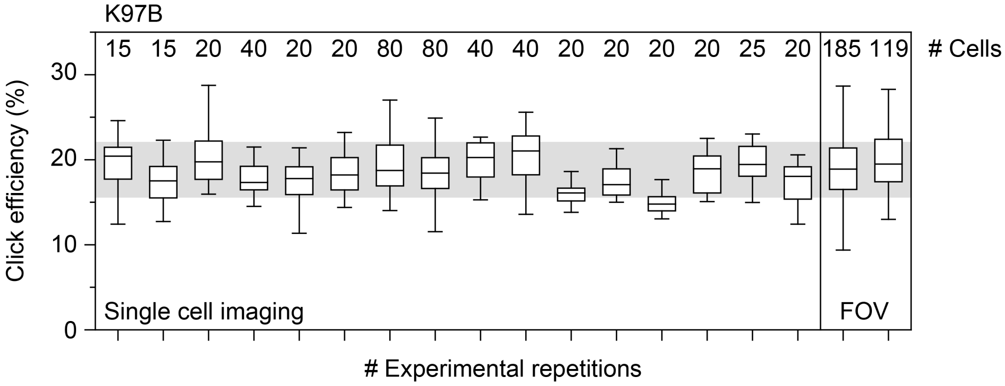

Supplementary Fig. 19. Quantification of click efficiency (CE) by brightness-calibrated ratiometric imaging (BCRI) is robust. Cells expressing K97B were click-labeled with Alexa568-tet1, imaged and analyzed with BCRI. Over the course of the project, CE turned out to be a highly reproducible parameter (CE = 19% ± 3%, mean ± SD, grey shading). Since CE was independent of receptor expression levels, manual selection of smaller subsets and automatic processing of a larger field of view (FOV) produced the same population averages. Box-and-whisker plots indicate first and third quartile (box), median (horizontal line), and 1.5 times the interquartile range (whiskers). The number of cells measured for each condition is stated above. Source data are provided as a Source Data file.

Supplementary Figure 20 Steiert, Schultz et al.

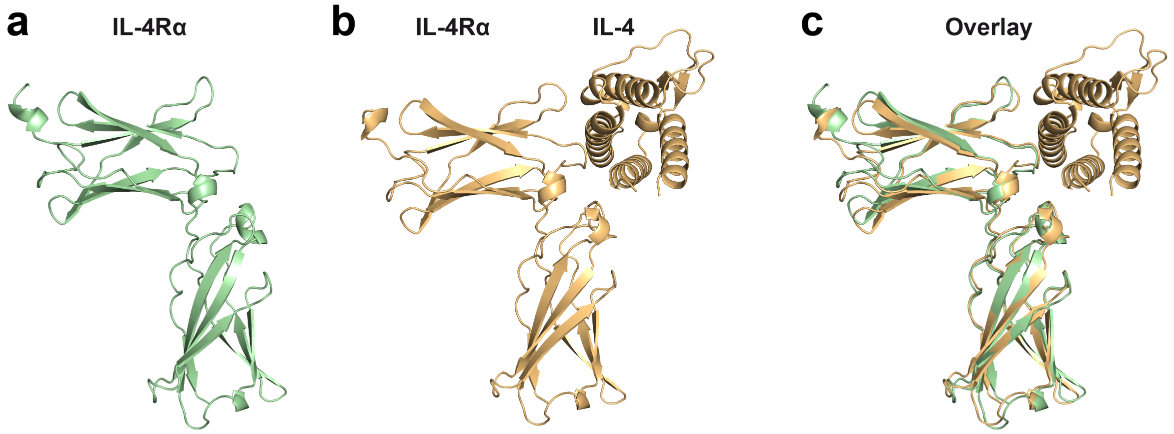

Supplementary Fig. 20. Molecular dynamics simulations based on crystal structures of IL-4Rα. Average structures of the extracellular IL-4Rα domains (PDB: 3BPN) as derived from 250 ns trajectories in water (**a**) devoid of or (**b**) including IL-4. (**c**) Overlay of the non-occupied (pale green) and occupied (pale yellow) receptor conformations to highlight structural differences, most pronounced within the protruding loops. Source data are provided as a Source Data file.

Supplementary Figure 21 Steiert, Schultz et al.

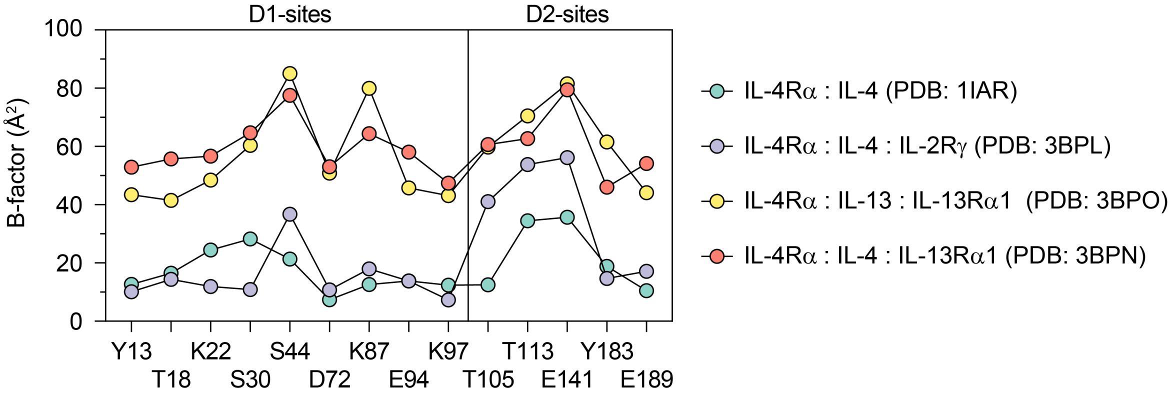

Supplementary Fig. 21. Backbone fluctuations of BCNK insertion sites from crystallographic data. Full isotropic B-factors potentially reflect the mobility of the Cα atoms of corresponding BCNK junctions in the protein backbone (mature numbering; Uniprot P24394-1). Values were taken from the databank of PDB files with consistent B-factors (BDB) [4] for the published crystal structures (PDBs: 1IAR, 3BPL, 3BPO, 3BPN) [5, 6]. Source data are provided as a Source Data file.

Supplementary Figure 22 Steiert, Schultz et al.

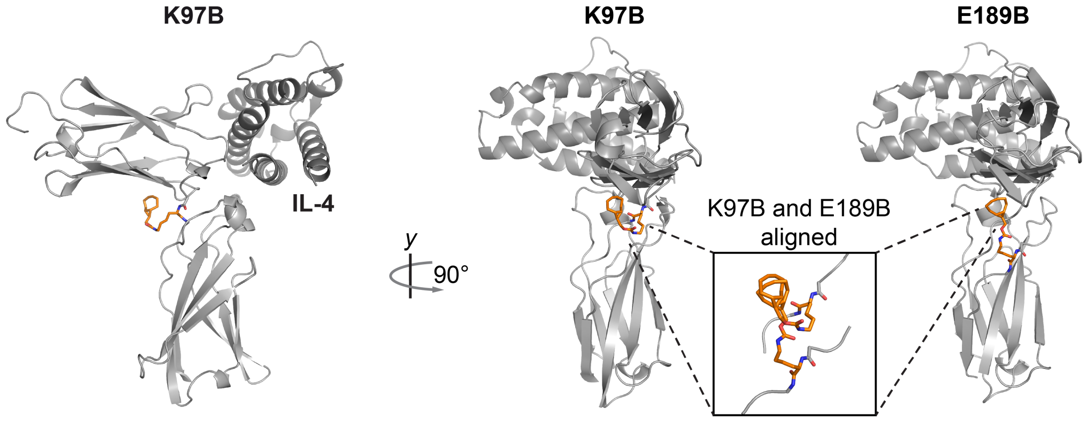

Supplementary Fig. 22. Structural relationship between chemical environment and click efficiency (CE). Average structures with subsequent minimization of simulated (250 ns) receptor mutants (PDB: 3BPN) bearing BCNK (representation as "sticks", orange) at indicated positions. K97B and E189B represent receptor mutants with maximum and minimum CE among the entire set of mutants (*cf.* Fig. 4a). Note the close proximity of the BCNK moieties in the aligned average structures of K97B and E189B (zoomed-in). Source data are provided as a Source Data file.

Supplementary Figure 23 Steiert, Schultz et al.

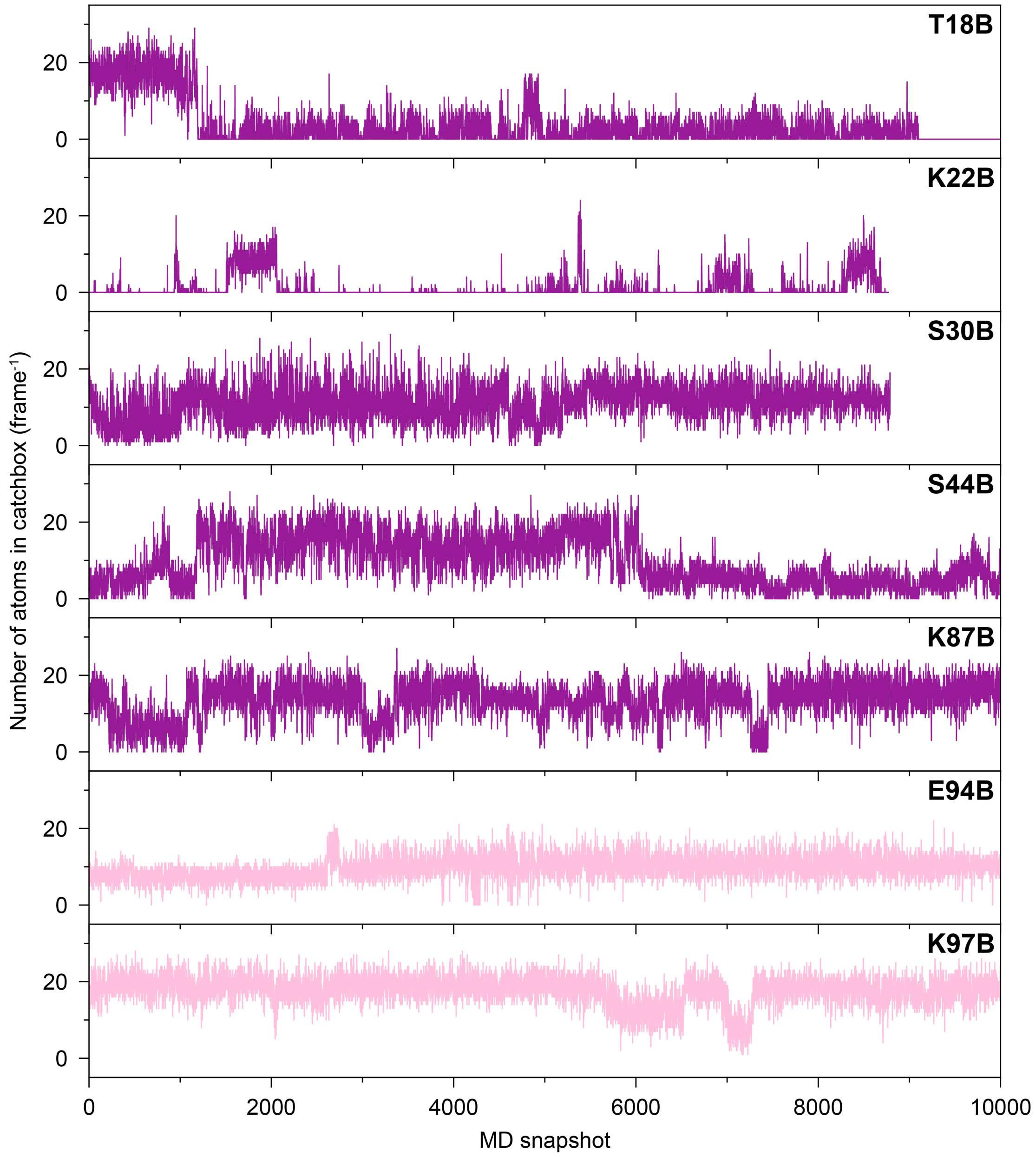

Supplementary Fig. 23. Environmental contacts of BCNK from MD simulations. Total number of IL-4Rα/IL4 atoms identified in the catchbox around the reactive center of the BCNK moiety for all simulated receptor mutants (color code according to Fig. 1a) along the trajectory. The time interval between MD snapshots is 25 ps. Source data are provided as a Source Data file.

Supplementary Figure 24 Steiert, Schultz et al.

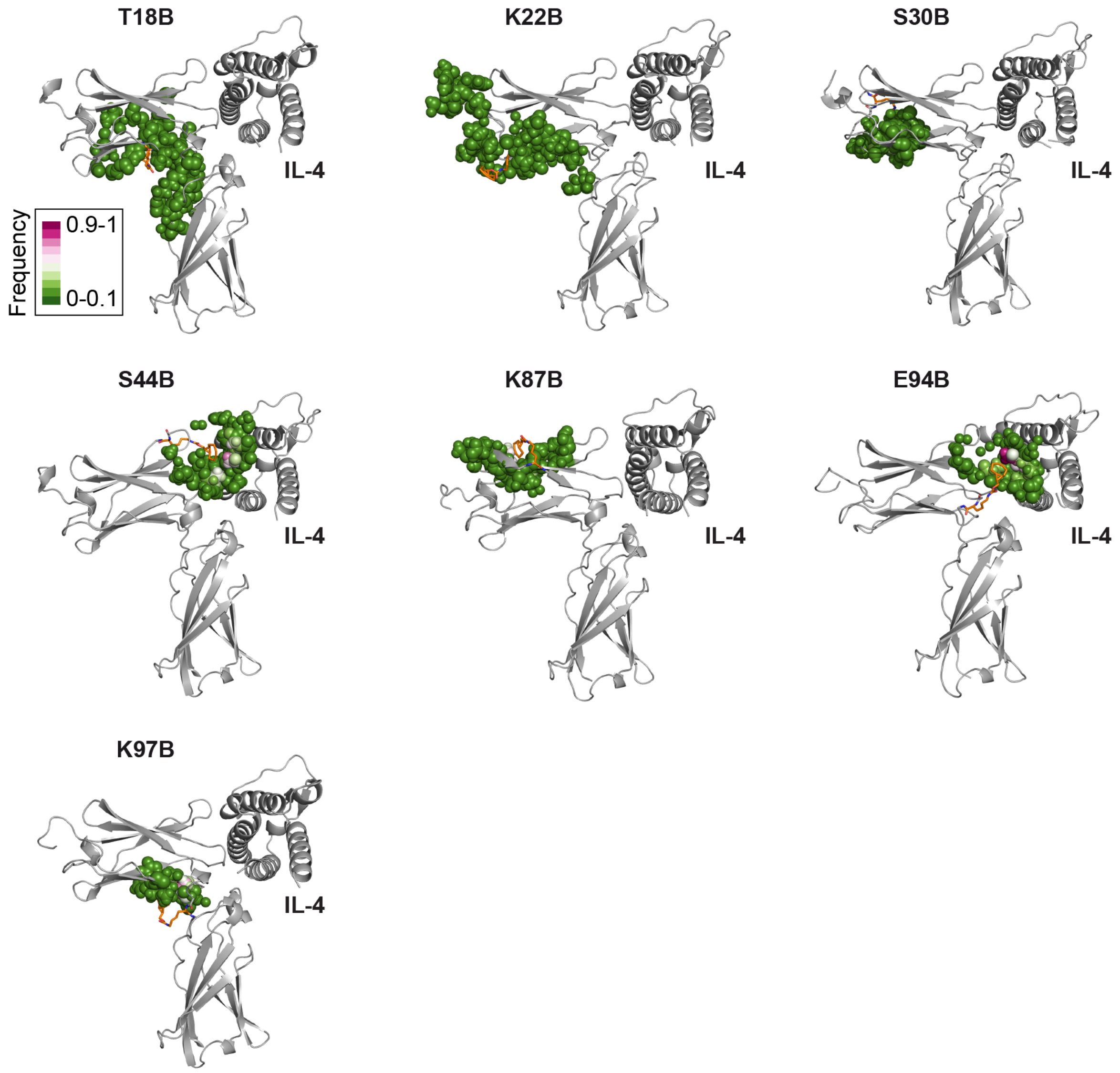

Supplementary Fig. 24. BCNK outreach during MD simulations. Average structures of receptor mutants (PDB: 3PBN; contact region as 'spheres', color-ramp from green to magenta) representing 250 ns MD simulations with subsequent minimization bearing the reactive ncAA BCNK ('sticks', orange) at indicated positions. Note the remarkable size of the protein surface from where contacts have been made. Source data are provided as a Source Data file.

Supplementary Figure 25 Steiert, Schultz et al.

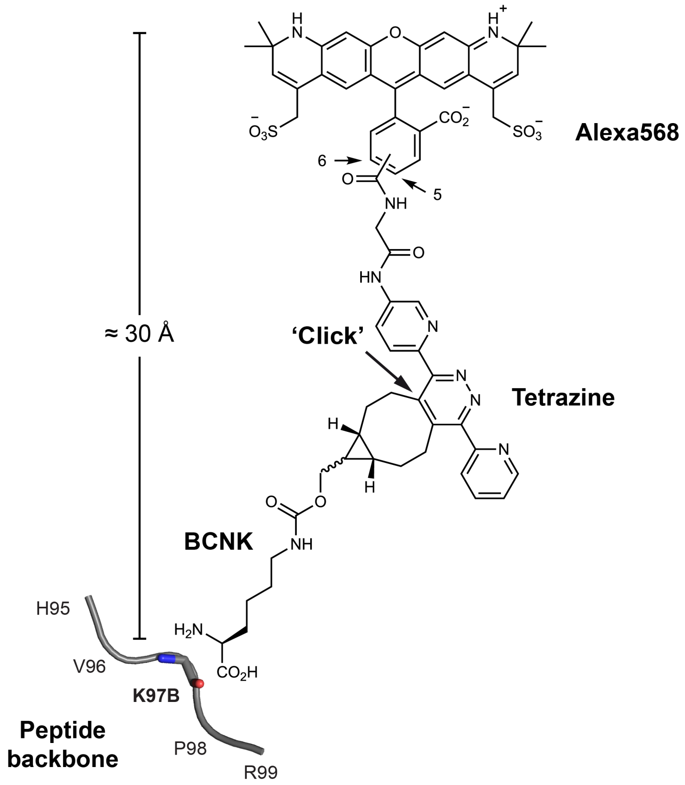

Supplementary Fig. 25. Dimension of the BCNK-tetrazine-dye-conjugate. Schematic representation of the molecular assembly conjugated to the peptide backbone of IL-4Rα* after click labeling. The example illustrates site-specific labeling of receptor mutant K97B with Alexa568-tet1. With a span of roughly 30 Å, the fluorophore-tetrazine-BCNK conjugate is of similar size as the extracellular domains of the IL-4Rα chain itself.

Supplementary Figure 26 Steiert, Schultz et al.

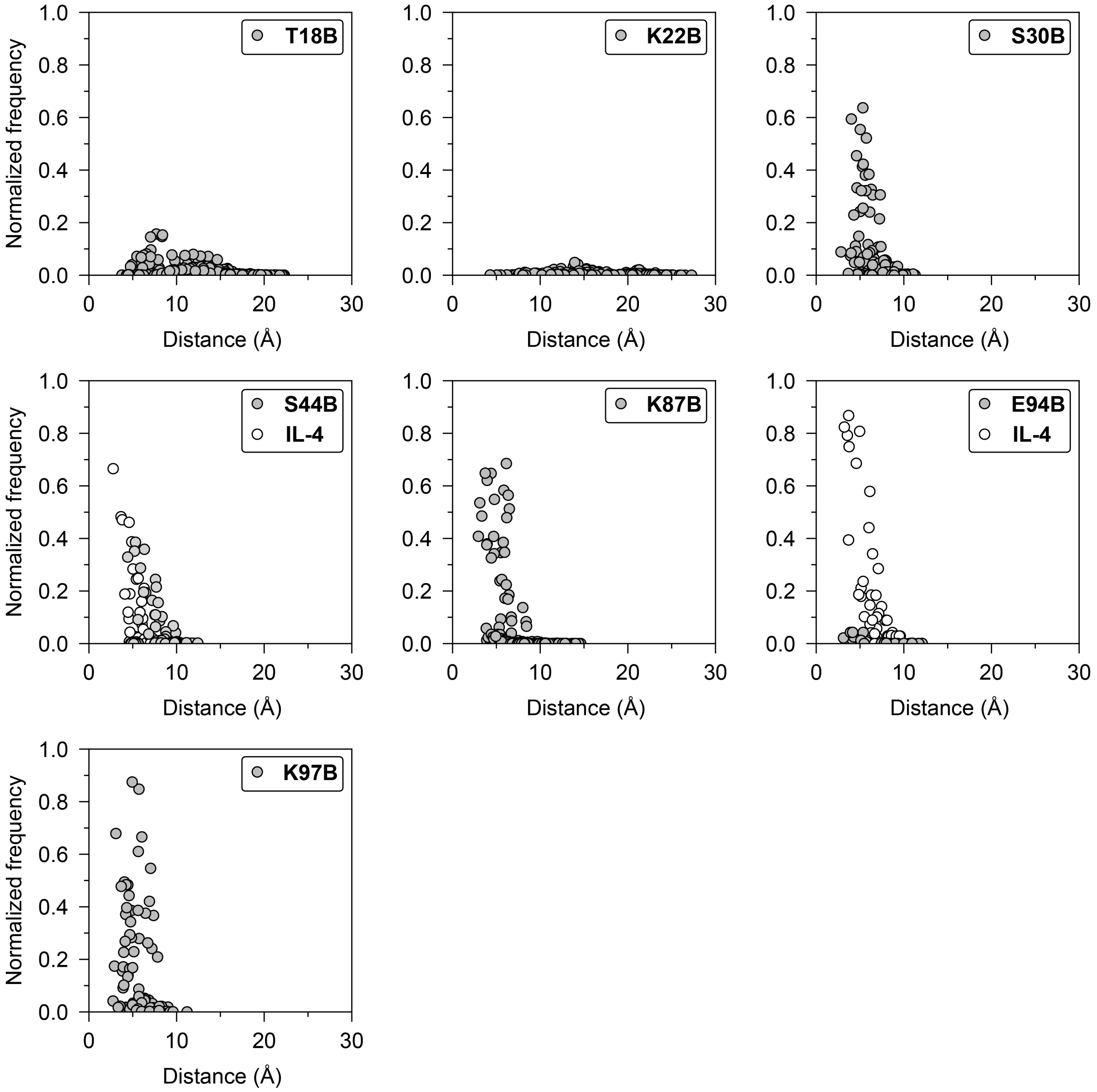

Supplementary Fig. 26. Physical distance of atoms making contact with BCNK. Distances were measured between sampled atoms and the center of the alkyne bond of BCNK in average structures representing 250 ns MD simulations with subsequent minimization. Frequencies were normalized by the number of simulated frames (~10,000). In most receptor mutants, due to specific interactions with neighboring hotspots, the contact area was confined to a radius of ~10 Å, whereas in the case of T18B and K22B the spatial range of BNCK movement was exceptionally large. Source data are provided as a Source Data file.

Supplementary Figure 27 Steiert, Schultz et al.

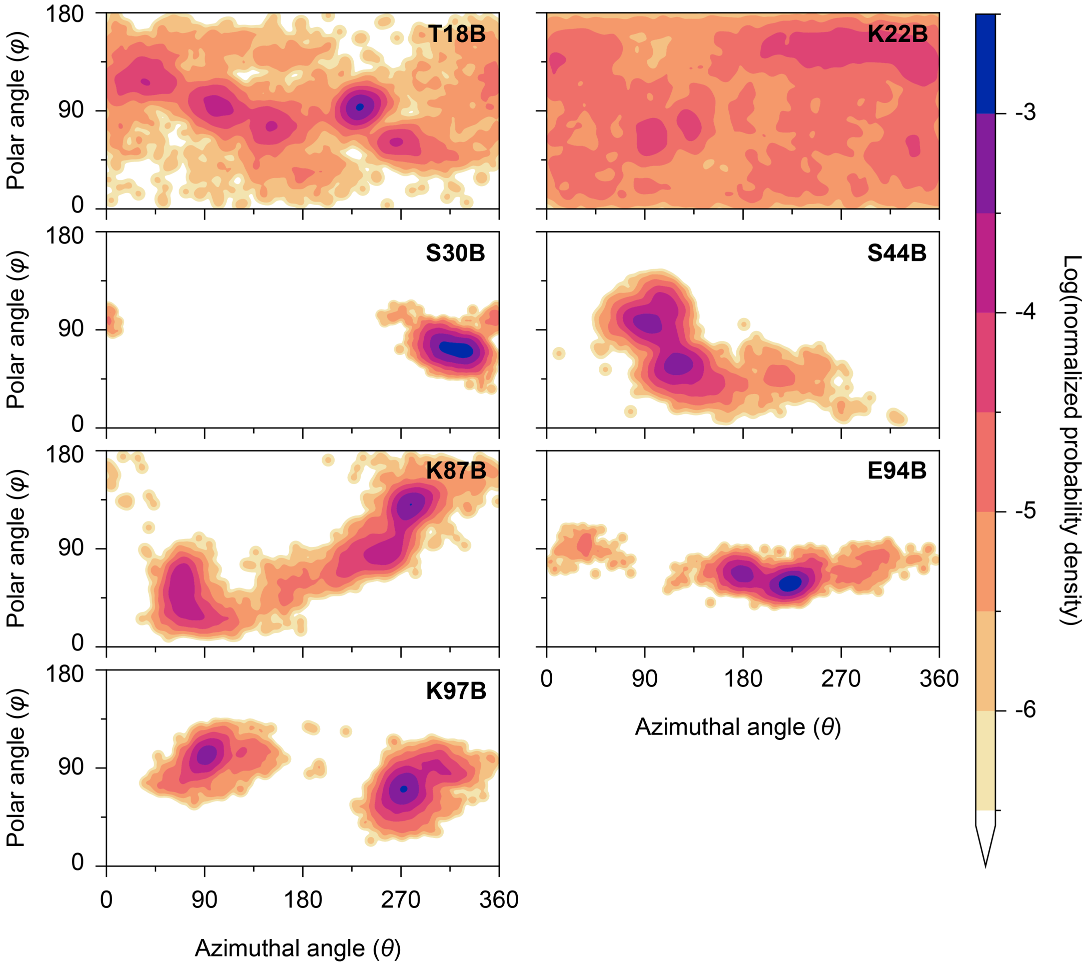

Supplementary Fig. 27. Rotary motion of the BCNK's ring. Probability distribution of ring orientations in spherical coordinates from 250 ns MD trajectories of BCNK-bearing receptor mutants. Polar and azimuthal angles are defined by the normal vector perpendicular to the plane of the ring. To visualize the distribution of angles, two-dimensional kernel density estimations were computed and plotted as filled contours with logarithmically-spaced levels. Probability densities below $10^{-6.5}$ are shown as white background. Source data are provided as a Source Data file.

Supplementary Figure 28 Steiert, Schultz et al.

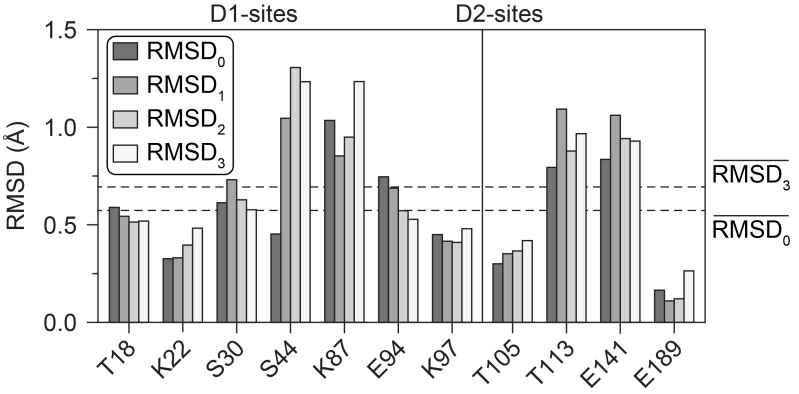

Supplementary Fig. 28. Ligand-dependent variability of the protein backbone of BCNK insertion sites. Root-mean-square deviations of the Cα atom with none ($RMSD_0$), one ($RMSD_1$), two ($RMSD_2$), or three ($RMSD_3$) adjacent Cα atoms along the protein backbone were calculated for indicated amino acid positions (mature numbering; Uniprot P24394-1) in crystal structures in the IL-4 occupied (PDB: 3BPN; IL-4Rα:IL-4:IL-13Rα1) and IL-13-occupied (PDB: 3BPO; IL-4Rα:IL-13:IL-13Rα1) state. The latter served as a proxy for a "non-occupied" IL-4Rα chain [6, 7]. The average RMSDs of all positions for the Cα atom with adjacent carbon atoms are indicated by dashed lines ($\overline{\mathrm{RMSD}_0} = 0.6$ Å and $\overline{\mathrm{RMSD}_3} = 0.7$ Å). Source data are provided as a Source Data file.

Supplementary Figure 29 Steiert, Schultz et al.

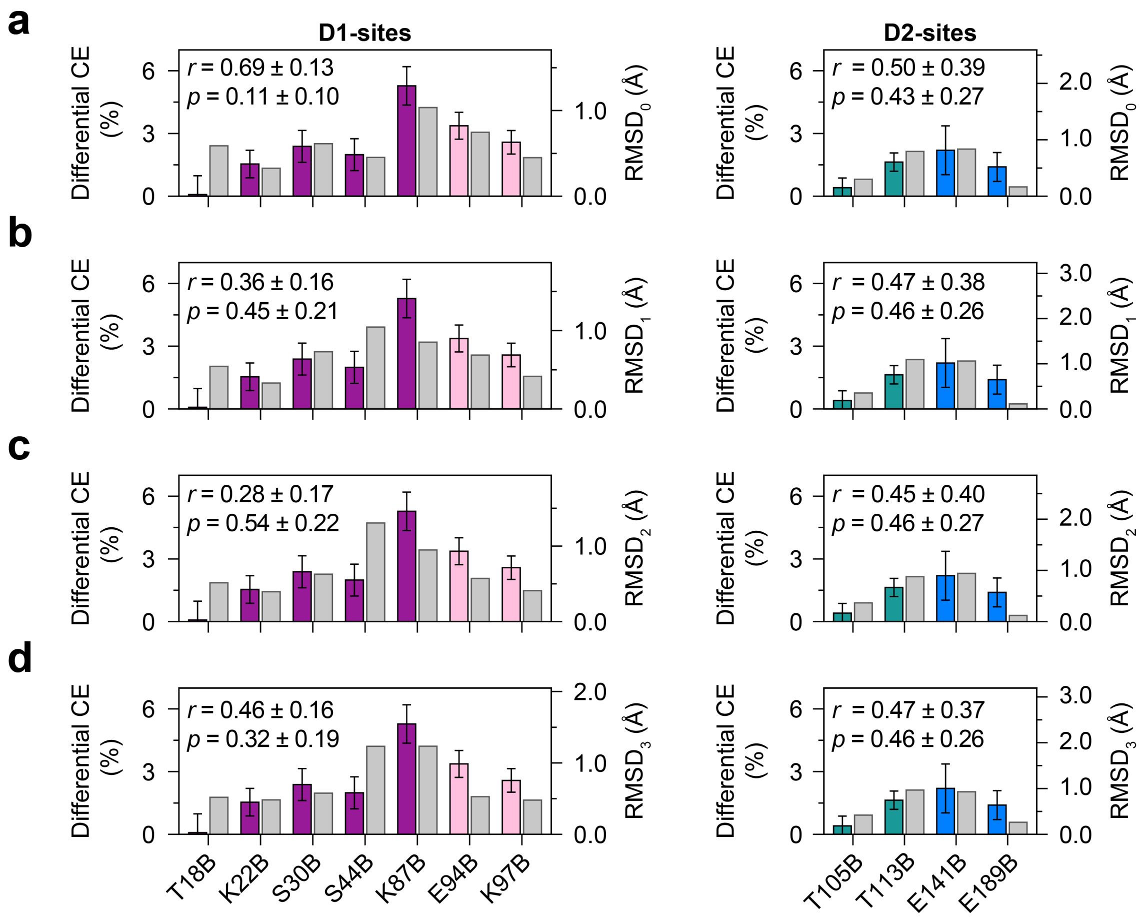

Supplementary Fig. 29. Correlation between ligand-dependent structural displacement and ligand-induced change in click efficiency. Correlation analysis of differential CE in absolute values measured for the indicated receptor mutants (color code according to Fig. 1a; mature numbering; Uniprot P24394-1) with ligand-dependent protein backbone deformations (RMSD, Supplementary Fig. 28) as quantified from superimposed crystal structures (PDB: 3BPN, 3BPO). RMSD values slightly increase depending on how wide the frame of Cα atoms was chosen: (**a**) only the BCNK junction itself is included ($RMSD_0$), or (**b**) one ($RMSD_1$), (**c**), two ($RMSD_2$), and (**d**) three ($RMSD_3$) adjacent Cα atoms. The limits of the second y-axis (right-hand side) were adjusted such that the average spread of differential CE and RMSD are identical for each subplot. For both domains, the corresponding Pearson correlation coefficient (*r*, top right corner) is maximized when only the BCNK-junction was considered ($RMSD_0$). Note that the dependence on adjacent Cα atoms was more pronounced in the flexible D1 domain. Only two BCNK insertion sites appeared to represent outliers: T18B for which a destructive conformation was sensed by ligand binding assays and E189B at the center position of the conserved WSXWS-motif of the activation loop. Bars and error bars are defined as mean ± SEM of CE differences in the presence and absence of IL-4 (see Supplementary Table 5 for precise *n* values). Error was estimated by uncertainty propagation assuming independent variables. Source data are provided as a Source Data file.

Supplementary Tables

Supplementary Table 1. Descriptive statistics of the BCNK titration assay.

BCNK [μM]	# Cells	Mean (μm^{-2})	SEM (μm^{-2})	Min (μm^{-2})	25% percentile (μm^{-2})	Median (μm^{-2})	75% percentile (μm^{-2})	Max (μm^{-2})
15.625	417	4.68	2.55	2.26	3.10	3.99	5.42	9.51
31.25	492	10.38	4.98	4.97	7.32	8.66	12.36	19.64
62.5	444	34.56	25.06	4.32	15.00	31.28	53.68	68.92
125	441	54.42	23.69	26.39	41.01	50.46	61.29	99.52
250	426	80.88	46.79	29.87	49.81	77.30	96.30	166.76
500	447	86.33	35.23	47.82	68.87	72.89	103.60	138.63
1000	479	90.47	40.39	20.78	80.57	82.58	107.11	154.59
2000	458	90.55	47.84	32.27	60.21	77.78	116.17	171.03

Supplementary Table 2. Descriptive statistics of the GCE expression assay.

	# Cells	Mean (μm^{-2})	Standard deviation (μm^{-2})	Min (μm^{-2})	25% percentile (μm^{-2})	Median (μm^{-2})	75% percentile (μm^{-2})	Max (μm^{-2})
IL-4Rα*	1304	612.04	791.50	0.73	44.67	274.38	890.34	4610.18
Mock	744	1.10	0.62	0.11	0.71	0.96	1.34	4.98
Y13B	844	60.81	196.47	0.26	2.35	6.43	31.65	2166.48
T18B	735	12.72	53.89	0.14	1.16	1.96	4.34	840.04
K22B	748	17.64	71.86	0.10	1.21	2.12	5.36	977.60
S30B	799	79.18	233.43	0.32	2.37	5.94	31.12	2049.81
S44B	774	24.42	81.98	0.22	1.36	2.45	7.74	984.24
D72B	637	74.18	240.21	0.30	2.33	5.61	28.93	2175.42
K87B	972	109.25	312.72	0.14	2.55	8.38	50.01	3526.41
E94B	756	251.65	490.60	0.48	10.57	42.30	215.93	3260.74
K97B	909	119.28	328.15	0.45	3.69	11.28	60.07	3037.03
K97(-)	783	1.89	2.47	0.11	0.83	1.27	1.98	33.61
T105B	826	61.83	166.50	0.08	2.86	7.73	35.79	2180.65
T113B	756	148.71	349.79	0.24	4.72	19.15	101.62	3176.24
E141B	728	84.54	193.58	0.18	3.93	14.60	70.39	2459.01
Y183B	800	61.03	192.69	0.28	2.27	6.02	27.25	2120.09
E189B	667	11.64	29.39	0.16	1.35	2.47	7.08	367.77

Supplementary Table 3. Descriptive statistics of receptor occupancy at 10 nM IL-4-ATTO647N.

	# Cells	Mean (%)	Standard deviation (%)	Min (%)	25% percentile (%)	Median (%)	75% percentile (%)	Max (%)
IL-4Rα*	406	99.41	8.21	70.05	94.72	99.03	104.56	129.48
Lyn-eGFP	37	0.01	0.12	-0.18	-0.04	-0.02	0.02	0.60
Y13B	232	0.06	0.74	-2.39	-0.13	0.09	0.34	3.51
T18B	250	61.49	16.57	9.62	52.64	63.21	71.83	123.20
K22B	212	95.27	12.02	57.06	88.55	95.88	102.16	127.77
S30B	181	83.19	15.03	29.25	74.63	83.94	92.80	126.04
S44B	195	62.16	11.07	28.57	55.56	61.31	68.03	97.33
D72B	87	0.34	0.37	0.00	0.12	0.23	0.46	2.63
K87B	377	86.42	12.05	47.97	78.77	86.18	93.97	123.02
E94B	359	91.11	12.50	57.14	82.64	91.68	99.59	125.49
K97B	722	83.90	14.67	15.53	76.14	84.38	93.04	128.58
T105B	579	76.71	18.43	0.35	65.15	78.74	88.50	125.34
T113B	352	77.69	9.83	51.68	70.72	77.86	84.10	108.92
E141B	429	50.18	26.23	8.86	27.83	43.65	71.09	113.19
Y183B	287	17.38	5.17	3.13	14.35	16.64	19.35	57.49
E189B	335	37.42	14.73	1.17	28.41	37.18	47.06	80.76

Supplementary Table 4. Descriptive statistics of receptor occupancy at 3 nM IL-4-ATTO647N.

	# Cells	Mean (%)	Standard deviation (%)	Min (%)	25% percentile (%)	Median (%)	75% percentile (%)	Max (%)
IL-4Rα*	342	99.39	8.46	61.92	94.33	99.98	105.28	118.41
Lyn-eGFP	183	0.04	0.10	-0.05	0.01	0.02	0.05	1.02
Y13B	138	0.14	0.24	-0.34	0.04	0.09	0.16	1.27
T18B	207	64.52	11.89	36.79	56.15	64.65	73.55	94.43
K22B	82	72.33	11.91	22.83	66.66	73.16	78.12	100.14
S30B	249	68.34	14.08	33.39	58.89	68.36	78.29	104.86
S44B	80	42.94	12.95	14.99	34.17	42.39	51.00	77.67
D72B	201	0.27	0.31	-0.06	0.11	0.17	0.29	2.33
K87B	227	69.68	10.98	41.57	62.06	69.42	77.22	118.58
E94B	200	56.67	12.40	24.30	49.67	58.57	64.90	88.62
K97B	133	63.39	12.29	33.04	56.18	63.77	71.94	91.38
T105B	173	68.49	16.38	31.79	55.23	69.34	80.98	104.91
T113B	252	54.23	9.63	2.36	47.92	54.69	60.58	75.95
E141B	155	32.62	22.44	6.04	13.16	24.25	51.53	87.11
Y183B	169	4.70	1.31	2.23	3.77	4.53	5.57	8.83
E189B	70	21.80	10.34	7.16	13.90	19.65	26.97	50.16

Supplementary Table 5. Descriptive statistics for the click efficiency of receptor mutants in the absence (-) and presence (+) of IL-4 ligand.

	IL-4	*n*	# Cells	Mean (%)	SEM (%)	Min (%)	25% percentile (%)	Median (%)	75% percentile (%)	Max (%)
Y13B	-	3	60	7.32	0.10	5.07	6.76	7.23	7.84	9.03
T18B	-	5	90	13.14	0.25	8.23	11.38	13.26	15.17	18.82
T18B	+	2	30	13.22	0.86	7.35	10.47	13.92	15.58	17.47
K22B	-	6	115	14.72	0.33	6.01	12.46	14.73	17.20	25.44
K22B	+	2	35	13.18	0.57	8.55	10.93	12.54	14.39	21.77
S30B	-	5	95	13.27	0.31	6.43	11.07	12.95	14.94	23.94
S30B	+	2	35	10.89	0.70	7.14	9.11	10.54	12.29	18.20
S44B	-	5	149	12.17	0.24	5.41	10.13	12.25	13.79	19.08
S44B	+	2	90	14.15	0.72	7.65	11.60	13.42	16.95	22.15
D72B	-	3	60	17.21	0.26	13.67	16.00	16.91	18.78	22.79
K87B	-	6	114	11.68	0.30	4.74	10.18	11.51	13.07	21.70
K87B	+	2	35	6.40	0.87	2.31	5.05	6.75	7.82	9.77
E94B	-	6	110	14.98	0.27	8.78	13.10	14.86	17.02	25.25
E94B	+	5	90	11.61	0.58	5.45	9.03	12.07	13.68	18.20
K97B	-	18	799	18.82	0.12	8.58	16.52	18.61	21.09	28.75
K97B	+	6	149	16.24	0.55	8.40	14.25	16.13	18.63	24.56
T105B	-	5	95	10.83	0.20	6.03	9.45	11.01	12.36	14.32
T105B	+	4	75	10.42	0.42	2.50	9.31	10.75	12.19	15.61
T113B	-	5	88	8.30	0.18	4.55	7.25	8.11	9.22	12.59
T113B	+	2	30	6.67	0.40	4.81	5.66	6.31	7.27	10.11
E141B	-	6	405	9.19	0.12	0.84	8.05	9.30	10.44	16.65
E141B	+	3	926	7.00	1.16	0.54	5.94	7.15	8.10	13.02
Y183B	-	3	90	8.15	0.23	2.77	6.88	8.08	9.54	14.10
E189B	-	3	59	5.55	0.18	2.45	4.81	5.67	6.31	8.31
E189B	+	2	80	6.95	0.67	4.85	6.33	7.03	7.62	8.55

Supplementary Table 6. Frequency of amino acids sampled in catchbox analysis for different receptor mutants.

	T18B	K22B	S30B	S44B	K87B	E94B	K97B	Total
ARG	4331	0	0	930	85499	15	106	90881
GLY	1	611	4261	41376	5	39621	0	85875
LEU	9990	114	5	2156	32029	39665	24	83983
SER	3963	0	0	615	0	44	76574	81196
ILE	5858	0	1976	0	0	890	46478	55202
GLU	2666	2363	42691	0	4	0	0	47724
ALA	325	8	4	27164	246	2683	0	30430
VAL	173	443	10980	5565	0	28	8660	25849
ASN	12	751	12472	1443	0	5196	0	19874
ASP	3376	0	0	4879	55	76	5818	14204
THR	3	1213	1	87	1368	7446	3634	13752
CYS	82	92	13312	0	180	0	9	13675
TRP	20	349	19	3935	3516	1127	0	8966
PRO	373	1594	6281	0	8	0	0	8256
HIE	169	1688	6	0	5	0	2637	4505
GLN	0	555	0	221	228	90	0	1094
TYR	253	0	0	0	12	0	357	622
MET	3	505	2	0	0	0	0	510
LYS	246	92	0	111	0	9	0	458
PHE	0	0	0	18	0	10	5	33

Supplementary Table 7. Partial solvation free energy contributions of IL-4Rα* and BCNK-bearing receptor mutants including the IL-4 ligand. Values represent mean ± SEM of 8–10 MD-derived average structures.

	n	Cavitation Free Energy ΔG^{cav} (kcal/mol)	Polarization Free Energy ΔG^{pol} (kcal/mol)	Dispersion Free Energy ΔG^{disp} (kcal/mol)	Net Solvation Free Energy G^{solv} (kcal/mol)
IL-4Rα*	10	590.59 ± 0.28	-1951.09 ± 19.12	-511.83 ± 0.67	-1872.33 ± 18.96
T18B	9	594.96 ± 0.35	-1963.18 ± 13.71	-509.67 ± 1.16	-1877.89 ± 14.15
K22B	9	593.11 ± 0.29	-1974.04 ± 14.40	-515.06 ± 0.66	-1895.99 ± 13.96
S30B	8	595.33 ± 0.29	-2058.18 ± 13.52	-512.74 ± 1.11	-1975.59 ± 13.22
S44B	10	594.03 ± 0.36	-2008.00 ± 17.01	-513.12 ± 0.94	-1927.09 ± 17.10
K87B	10	594.26 ± 0.36	-2152.52 ± 20.53	-510.90 ± 1.50	-2069.16 ± 21.64
E94B	10	595.20 ± 0.31	-2047.56 ± 8.68	-508.67 ± 0.81	-1961.03 ± 8.43
K97B	10	594.65 ± 0.20	-1922.44 ± 18.13	-508.93 ± 0.91	-1836.72 ± 18.14

Supplementary Table 8. RMSD of the protein backbone for crystal structures in the IL-13- and IL-4-occupied state (PDBs: 3BPO, 3BPN). Subscripts indicate the number of neighboring Cα atoms included in the calculation of the RMSD.

	$RMSD_0$ (Å)	$RMSD_1$ (Å)	$RMSD_2$ (Å)	$RMSD_3$ (Å)
T18	0.589	0.544	0.514	0.519
K22	0.326	0.331	0.396	0.483
S30	0.613	0.731	0.628	0.577
S44	0.453	1.046	1.306	1.233
K87	1.035	0.853	0.949	1.234
E94	0.746	0.688	0.572	0.528
K97	0.450	0.416	0.410	0.480
T105	0.300	0.352	0.366	0.419
T113	0.794	1.093	0.878	0.967
E141	0.835	1.061	0.942	0.929
E189	0.165	0.110	0.121	0.264

Supplementary References

1. Lang, K. et al. Genetically encoded norbornene directs site-specific cellular protein labelling via a rapid bioorthogonal reaction. *Nat. Chem.* **4**, 298-304 (2012).
2. Lang, K. et al. Genetic Encoding of bicyclononynes and trans-cyclooctenes for site-specific protein labeling in vitro and in live mammalian cells via rapid fluorogenic Diels-Alder reactions. *J. Am. Chem. Soc.* **134**, 10317-10320 (2012).
3. Weidemann, T., Hofinger, S., Muller, K. & Auer, M. Beyond Dimerization: A Membrane-dependent Activation Model for Interleukin-4 Receptor-mediated Signalling. *J. Mol. Biol.* **366**, 1365-1373 (2007).
4. Touw, W.G. & Vriend, G. BDB: databank of PDB files with consistent B-factors. *Protein. Eng. Des. Sel.* **27**, 457-462 (2014).
5. Hage, T., Sebald, W. & Reinemer, P. Crystal structure of the interleukin-4/receptor alpha chain complex reveals a mosaic binding interface. *Cell* **97**, 271-281 (1999).
6. LaPorte, S.L. et al. Molecular and structural basis of cytokine receptor pleiotropy in the interleukin-4/13 system. *Cell* **132**, 259-272 (2008).
7. Gandhi, H. et al. Dynamics and interaction of interleukin-4 receptor subunits in living cells. *Biophys. J.* **107**, 2515-2527 (2014).
