# Supplementary material for: Insights into receptor structure and dynamics at the surface of living cells
Source: Nat Commun. 2023 Mar 22;14:1596. doi: 10.1038/s41467-023-37284-4 (PMC10033668; doi:10.1038/s41467-023-37284-4)
Supplement: Supplementary file 2 — Description of Additional Supplementary Files [file 41467_2023_37284_MOESM2_ESM.pdf]

Title: Supplementary Software
Description: Computational Biology Toolkit (AMBER version 20) providing material and code for Molecular Dynamics simulations and post-processing of trajectories. Examples of BCNK incorporation into a given structure (subfolder a), minimization, equilibration and production of MD simulations (subfolder b), the catchbox analysis (subfolder c), computation of MD-derived average structures (subfolder d), extraction of the orientation of the BCNK ring system (subfolder e), and the calculation of B-factors (subfolder f) are included. Further details are described in the README file.

Title: Supplementary Movie 1.
Description: Conformational dynamics of the IL-4Rα/IL-4 complex (PDB: 3BPN [http://doi.org/10.2210/pdb3BPN/pdb]) neglecting the IL-4 ligand part. The movie shows the conformational dynamics of the receptor in aqueous solution (water not shown) as observed during 250 ns of MD simulation. The activation loop, a crucial region for the activation of signal transduction, is highlighted in blue. The time course of the MD trajectory is represented as 360° view.

Title: Supplementary Movie 2.
Description: Conformational dynamics of the IL-4Rα/IL-4 complex (PDB: 3BPN [http://doi.org/10.2210/pdb3BPN/pdb]) including the IL-4 ligand. The movie shows the conformational dynamics of the complex in aqueous solution (water not shown) as observed during 250 ns of MD simulation. The activation loop, a crucial region for the activation of signal transduction, is highlighted in blue. The time course of the MD trajectory is represented as 360° view.

Title: Supplementary Movie 3.
Description: Conformational dynamics of the IL-4Rα/IL-4 complex (PDB: 3BPN [http://doi.org/10.2210/pdb3BPN/pdb]) containing single-point BCNK incorporation (orange) at position T18. The movie shows the conformational dynamics of the complex in aqueous solution (water not shown) as observed during 250 ns of MD simulation. The activation loop, a crucial region for the activation of signal transduction, is highlighted in blue. The time course of the MD trajectory is represented as 360° view.

Title: Supplementary Movie 4.
Description: Conformational dynamics of the IL-4Rα/IL-4 complex (PDB: 3BPN [http://doi.org/10.2210/pdb3BPN/pdb]) containing single-point BCNK incorporation (orange) at position K22. The movie shows the conformational dynamics of the complex in aqueous solution (water not shown) as observed during 250 ns of MD simulation. The activation loop, a crucial region for the activation of signal transduction, is highlighted in blue. The time course of the MD trajectory is represented as 360° view.

Title: Supplementary Movie 5.
Description: Conformational dynamics of the IL-4Rα/IL-4 complex (PDB: 3BPN

[http://doi.org/10.2210/pdb3BPN/pdb]) containing single-point BCNK incorporation (orange) at position S30. The movie shows the conformational dynamics of the complex in aqueous solution (water not shown) as observed during 250 ns of MD simulation. The activation loop, a crucial region for the activation of signal transduction, is highlighted in blue. The time course of the MD trajectory is represented as 360° view.

Title: Supplementary Movie 6.
Description: Conformational dynamics of the IL-4Rα/IL-4 complex (PDB: 3BPN [http://doi.org/10.2210/pdb3BPN/pdb]) containing single-point BCNK incorporation (orange) at position S44. The movie shows the conformational dynamics of the complex in aqueous solution (water not shown) as observed during 250 ns of MD simulation. The activation loop, a crucial region for the activation of signal transduction, is highlighted in blue. The time course of the MD trajectory is represented as 360° view.

Title: Supplementary Movie 7.
Description: Conformational dynamics of the IL-4Rα/IL-4 complex (PDB: 3BPN [http://doi.org/10.2210/pdb3BPN/pdb]) containing single-point BCNK incorporation (orange) at position K87. The movie shows the conformational dynamics of the complex in aqueous solution (water not shown) as observed during 250 ns of MD simulation. The activation loop, a crucial region for the activation of signal transduction, is highlighted in blue. The time course of the MD trajectory is represented as 360° view.

Title: Supplementary Movie 8.
Description: Conformational dynamics of the IL-4Rα/IL-4 complex (PDB: 3BPN [http://doi.org/10.2210/pdb3BPN/pdb]) containing single-point BCNK incorporation (orange) at position E94. The movie shows the conformational dynamics of the complex in aqueous solution (water not shown) as observed during 250 ns of MD simulation. The activation loop, a crucial region for the activation of signal transduction, is highlighted in blue. The time course of the MD trajectory is represented as 360° view.

Title: Supplementary Movie 9.
Description: Conformational dynamics of the IL-4Rα/IL-4 complex (PDB: 3BPN [http://doi.org/10.2210/pdb3BPN/pdb]) containing single-point BCNK incorporation (orange) at position K97. The movie shows the conformational dynamics of the complex in aqueous solution (water not shown) as observed during 250 ns of MD simulation. The activation loop, a crucial region for the activation of signal transduction, is highlighted in blue. The time course of the MD trajectory is represented as 360° view.
