# Supplementary material for: Insights into receptor structure and dynamics at the surface of living cells
Source: Nat Commun. 2023 Mar 22;14:1596. doi: 10.1038/s41467-023-37284-4 (PMC10033668; doi:10.1038/s41467-023-37284-4)
Supplement: Supplementary file 3 — Reporting Summary [file 41467_2023_37284_MOESM3_ESM.pdf]

nature portfolio

Corresponding author(s): Dr. Thomas Weidemann

Last updated by author(s): Mar 9, 2023

Reporting Summary

Nature Portfolio wishes to improve the reproducibility of the work that we publish. This form provides structure for consistency and transparency in reporting. For further information on Nature Portfolio policies, see our Editorial Policies and the Editorial Policy Checklist.

Statistics

For all statistical analyses, confirm that the following items are present in the figure legend, table legend, main text, or Methods section.

n/a	Confirmed	
☐	☒	The exact sample size (*n*) for each experimental group/condition, given as a discrete number and unit of measurement
☐	☒	A statement on whether measurements were taken from distinct samples or whether the same sample was measured repeatedly
☐	☒	The statistical test(s) used AND whether they are one- or two-sided<br>*Only common tests should be described solely by name; describe more complex techniques in the Methods section.*
☐	☒	A description of all covariates tested
☒	☐	A description of any assumptions or corrections, such as tests of normality and adjustment for multiple comparisons
☐	☒	A full description of the statistical parameters including central tendency (e.g. means) or other basic estimates (e.g. regression coefficient) AND variation (e.g. standard deviation) or associated estimates of uncertainty (e.g. confidence intervals)
☐	☒	For null hypothesis testing, the test statistic (e.g. *F*, *t*, *r*) with confidence intervals, effect sizes, degrees of freedom and *P* value noted<br>*Give P values as exact values whenever suitable.*
☒	☐	For Bayesian analysis, information on the choice of priors and Markov chain Monte Carlo settings
☒	☐	For hierarchical and complex designs, identification of the appropriate level for tests and full reporting of outcomes
☐	☒	Estimates of effect sizes (e.g. Cohen's *d*, Pearson's *r*), indicating how they were calculated

Our web collection on statistics for biologists contains articles on many of the points above.

Software and code

Policy information about availability of computer code

Data collection	ZEN microscopy software, blue edition, version 3.0 (Zeiss)
Data analysis	Microscopy images were analyzed with a custom-written Fiji (ImageJ, version 2.9.0/1.53t; Java 1.8.0_172) script available under the following repository: https://doi.org/10.17617/3.YDSJ2C.<br>Post-processing of imaging data and plotting was performed in Python (version 3.8.5) using the packages numpy (version 1.20.3), pandas (version 1.2.5), scipy (version 1.6.2), scikit-learn (version 0.24.2), and matplotlib (version 3.3.4). Custom code is available on request from the corresponding author.<br>Molecular dynamics simulations and post-processing were performed with the AMBER software suite (version 20) and its associated programs (antechamber, prepgen, sander, bcc, pmemd.MPI, pmemd.cuda, cpptraj), as well as with MOLDEN (version 5.9.2), GAUSSIAN (version 09), VMD (version 1.9), the SHAKE algorithm and POLCH (version 2.3). Materials and code are provided as Supplementary Software (SI_comp_bio.zip).

For manuscripts utilizing custom algorithms or software that are central to the research but not yet described in published literature, software must be made available to editors and reviewers. We strongly encourage code deposition in a community repository (e.g. GitHub). See the Nature Portfolio guidelines for submitting code & software for further information.

Data

Policy information about availability of data

All manuscripts must include a data availability statement. This statement should provide the following information, where applicable:

- Accession codes, unique identifiers, or web links for publicly available datasets
- A description of any restrictions on data availability
- For clinical datasets or third party data, please ensure that the statement adheres to our policy

Source data are provided with this paper. Raw microscopy data generated during the study are available on request due to the large file size, requests should be made to the corresponding author and will be answered within 2 weeks. The following publicly available datasets from the Protein Data Bank were used in the study: 1IAR [http://doi.org/10.2210/pdb1IAR/pdb], 3BPN [http://doi.org/10.2210/pdb3BPN/pdb], 3BPO [http://doi.org/10.2210/pdb3BPO/pdb], 3BPL [http://doi.org/10.2210/pdb3BPL/pdb]

Human research participants

Policy information about studies involving human research participants and Sex and Gender in Research.

Reporting on sex and gender	n/a
Population characteristics	n/a
Recruitment	n/a
Ethics oversight	n/a

Note that full information on the approval of the study protocol must also be provided in the manuscript.

Field-specific reporting

Please select the one below that is the best fit for your research. If you are not sure, read the appropriate sections before making your selection.

☒ Life sciences ☐ Behavioural & social sciences ☐ Ecological, evolutionary & environmental sciences

For a reference copy of the document with all sections, see nature.com/documents/nr-reporting-summary-flat.pdf

Life sciences study design

All studies must disclose on these points even when the disclosure is negative.

Sample size	Typically, at least 15 cells were measured for each condition to obtain a sufficiently robust readout.
Data exclusions	No data was excluded.
Replication	At least three independent experiments were performed for all measurements if not stated otherwise. All replicates were used since none deviated significantly.
Randomization	Cells were randomly assigned to different experimental groups during sample preparation.
Blinding	Blinding was not required as the properties of single-cell measurements are unknown prior to their analysis (except for edge cases).

Reporting for specific materials, systems and methods

We require information from authors about some types of materials, experimental systems and methods used in many studies. Here, indicate whether each material, system or method listed is relevant to your study. If you are not sure if a list item applies to your research, read the appropriate section before selecting a response.

Materials & experimental systems

n/a	Involved in the study	
x		Antibodies
	x	Eukaryotic cell lines
x		Palaeontology and archaeology
x		Animals and other organisms
x		Clinical data
x		Dual use research of concern

Methods

n/a	Involved in the study	
x		ChIP-seq
x		Flow cytometry
x		MRI-based neuroimaging

Eukaryotic cell lines

Policy information about cell lines and Sex and Gender in Research

Cell line source(s)	HEK293T (ATCC CRL-3216)
Authentication	Cells were kept at low passage and were not further authenticated.
Mycoplasma contamination	Cell line was tested negative by PCR for mycoplasma contamination
Commonly misidentified lines (See ICLAC register)	None
